# Evolvability of Drought Response in Four Native and Non-native Conifers: Opportunities for Forest and Genetic Resource Management in Europe

**DOI:** 10.3389/fpls.2021.648312

**Published:** 2021-07-08

**Authors:** Silvio Schueler, Jan-Peter George, Sandra Karanitsch-Ackerl, Konrad Mayer, Raphael Thomas Klumpp, Michael Grabner

**Affiliations:** ^1^Department of Forest Growth, Silviculture and Genetics, Austrian Research Centre for Forests BFW, Vienna, Austria; ^2^Department of Material Sciences and Process Engineering, Institute of Wood Technology and Renewable Resources, University of Natural Resources and Life Sciences (BOKU), Tulln an der Donau, Austria; ^3^Department of Forest- and Soil Sciences, Institute of Silviculture, University of Natural Resources and Life Sciences (BOKU), Vienna, Austria

**Keywords:** *Picea abies*, *Abies alba*, *Larix decidua*, *Pseudotsuga menziesii*, coefficient of additive genetic variation, repeatability, assisted migration and gene flow, tree breeding and improvement

## Abstract

Intraspecific genetic variation in drought response is expected to play an important role in determining the persistence of tree populations in global change as it (1) allows for spontaneous selection and local adaptation of tree populations, (2) supports assisted seed transfer of less-drought-sensitive provenance, and (3) enables the integration of drought-sensitivity traits into tree breeding. Estimating the potential of such adaptation options requires quantitative genetic knowledge of drought sensitivity across significant parts of species distributions and a comparative assessment of genetic variation within economically and ecologically important tree species. We quantified genetic variation within and among populations of four conifers growing within common garden experiments in the drought-prone eastern Austria. This region experienced three strong drought periods between 1980 and 2010 that resulted in significant reductions in radial growth. Among the four tested species, Douglas-fir revealed the highest resistance during drought and silver fir the best recovery after drought, while European larch and Norway spruce showed the lowest resistance. High genetic variation among populations and phenotypic stability across all three drought events was found for Norway spruce and silver fir, but not for the other species. Heritability and evolvability of drought traits, both approximated *via* genetic repeatability, revealed strong differences among populations of all four species. Repeatability and evolvability for resistance were highest in Norway spruce and, for recovery, highest in European larch. Our comparison indicates that the mean drought sensitivity of a species is not related to the intraspecific genetic variation in drought response. Thus, also highly drought-sensitive species, such as Norway spruce and European larch, harbor significant genetic variation in drought response within and among populations to justify targeted tree breeding, assisted gene flow, and supportive forest management to foster local adaptations to future conditions.

## Introduction

Increasing severity, duration, and higher frequency of drought events were identified as important consequences of climate change (Dai, 2014; Haslinger et al., 2016) and having a significant impact on forest ecosystems and the various services they provide (Allen et al., 2010). The reaction of trees includes short- and long-term growth reductions (Sarris et al., 2007), lower-tree vigor (Camarero et al., 2015), and reduced defense against insects and fungi (Cech and Tomiczek, 1996; Wermelinger et al., 2008) and, hence, higher tree mortality (Sala et al., 2010). Moreover, the observed increase of tree mortality and forest dieback as consequences of large-scale drought periods worldwide raised concerns about the role of forests as a carbon sink and the risks of positive feedback to rising CO_2_ concentrations (Breshears and Allen, 2002; Kurz et al., 2008).

To predict future tree mortality and the carbon balance of forest ecosystems as well as to develop management strategies that cope with frequent drought occurrence, a better understanding is needed (1) of the physiological limits and traits connected to drought tolerance of individual trees (Sohn et al., 2013; Bennett et al., 2015; Trujillo-Moya et al., 2018), (2) of the genetic diversity of traits related to drought resistance and avoidance, (3) of local adaptations and the adaptive capacity of single populations, for example, growing at marginal sites (Stojnic et al., 2018), and (4) water use strategies and hydraulic niches of tree species (Choat et al., 2012; Hochberg et al., 2018).

Short- and mid-term consequences of drought-induced mortality in climate change were suggested to be mitigated by planting more resistant tree species (Broadmeadow et al., 2005; Jandl et al., 2015) or management activities that foster tree vigor and drought tolerance (Sohn et al., 2016; Bradford and Bell, 2017). However, the long-term fate of forest tree populations depends, to a large degree, on their phenotypic plasticity and adaptive capacity to deal with the changing environmental conditions. These may include, for example, local adaptations available within marginal populations (Rose et al., 2009; Aranda et al., 2015), rare alleles associated with specific resistance traits (Thoen et al., 2017), or standing genetic variation (Barrett and Schluter, 2008; Fady et al., 2016).

A management concept to deal with the vulnerability of endangered populations at the warm temperature edge of species ranges and the planting of seed sources better adapted to future conditions is assisted gene flow (Aitken and Bemmels, 2016), often more generally termed as “assisted population migration.” In this concept, adaptive clines across a species range or populations with particular adaptive traits are being identified and transferred to promote the adaption of forest ecosystems to the new climate conditions. A precondition for such management is a decent knowledge of adaptive clines and the multiple traits that underlie local adaptations to climate conditions (Chevin et al., 2013). The sensitivity of tree growth to drought events was found to be related to future tree mortality (Cailleret et al., 2019; DeSoto et al., 2020) and might thus be used as an indicator for vigorous tree populations and individuals and as a trait to select future seed material. However, drought sensitivity might be based on various molecular (Trujillo-Moya et al., 2018), morphological (Rosner et al., 2014), or physiological characteristics (Martin-St. Paul et al., 2017), and the complexity of drought reaction challenges simple recommendations for assisted gene flow in trees (Isaac-Renton et al., 2018).

Another option to improve the resilience of forests to extreme drought conditions is the integration of drought sensitivity as an adaptive trait within ongoing tree improvement programs. So far, tree breeding in temperate and boreal conifers mainly considered productivity traits such as height growth, volume production, and stem characteristics or adaptive traits related to growth in cold environments, including frost hardiness, bud burst, or bud set (Hannerz et al., 1999). For such traits, detailed knowledge of quantitative genetic variation within and between populations, as well as genetic correlations among traits, is well-established (Howe et al., 2003; St. Clair, 2006) and integrated into ongoing tree improvement and assisted gene flow programs (Aitken and Hannerz, 2001; Hannerz and Ericsson, 2007; Berlin et al., 2016).

In the present study, we aim at testing for quantitative genetic variation in drought response among and within populations of four important conifers. Three species, Norway spruce (*Picea abies* [L.] Karst.), silver fir (*Abies alba* Mill.), and European larch (*Larix decidua* Mill.), are native to Central Europe and represent a major part of conifer forests in Alpine, temperate and boreal forest. In addition, Douglas-fir (*Pseudotsuga menziesii* [Mirbel] Franco), the most widespread non-native conifer planted in Europe, is considered an alternative tree species providing high productivity and resilience to climate change (Chakraborty et al., 2016). Genetic variation among populations is required to develop recommendations for assisted migration and gene flow, whereas genetic variation within populations is required for management-facilitated adaptation and tree breeding. Using provenance trials growing in the drought-prone East of Austria, we analyzed the effects of consecutive severe drought periods on individual tree growth, resistance, and recovery from drought. This allowed us to estimate the stability of drought reaction of different populations, i.e., the growth response of individual trees and populations during and after consecutive drought periods, the degree of genetic determination, and the ability of populations and species to respond to natural selection, termed as “evolvability.”

## Materials and Methods

### Trial Sites, Plant Material, and Sampling

**Figure 1:**
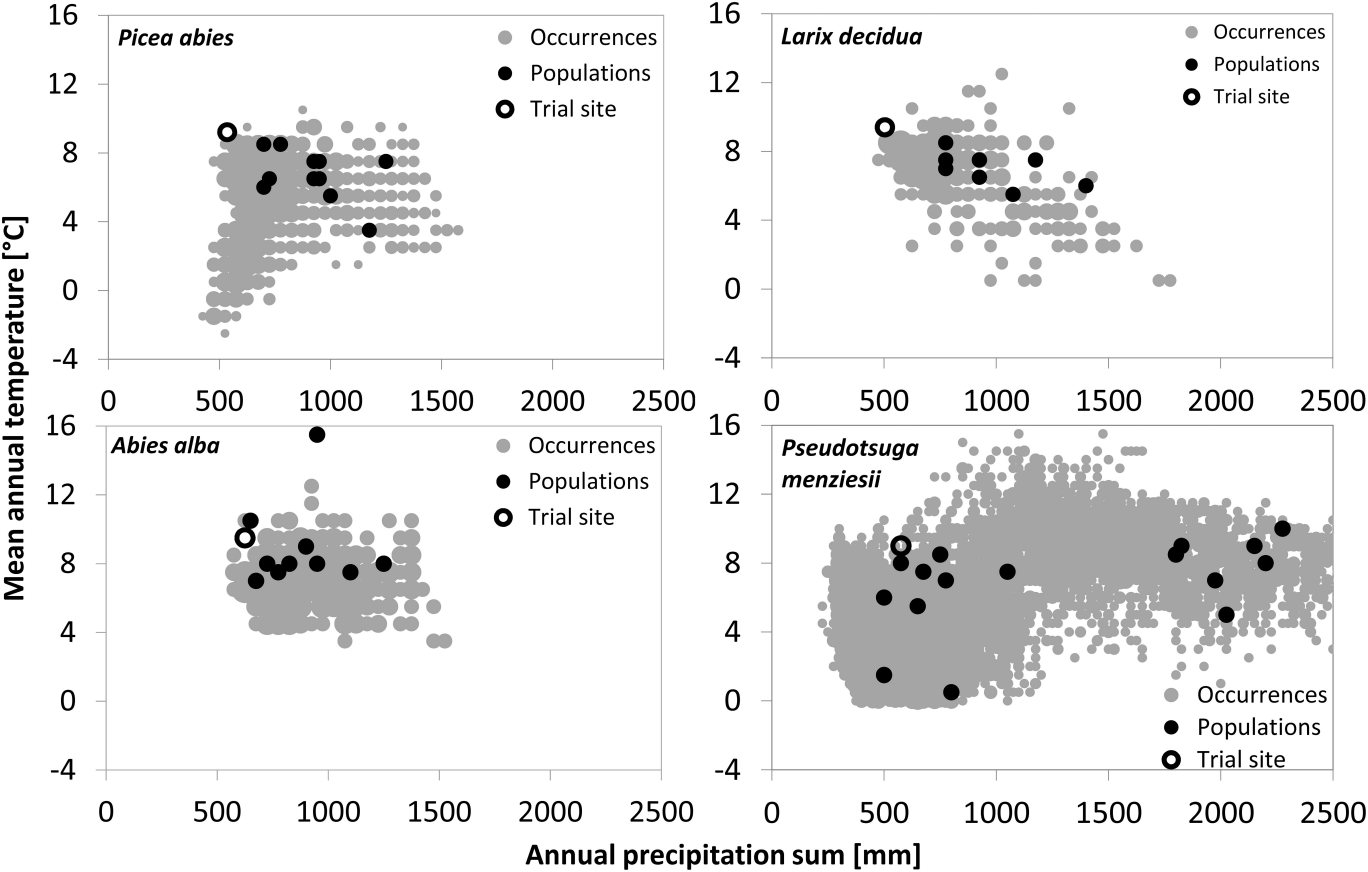
Climatic origin of the tested provenances and the four trial sites in relation to the overall climate niche of the respective conifers. Species occurrences were obtained from the ICP Forest Program Level 1 for the three European species and from Schroeder et al. (2010) for Douglas-fir. Climate data of species occurrences and provenance origin are based upon WorldClim climate data (Hijmans et al., 2005), whereas the trial site climate represents the mean climate from 1961 to 2011.

The trial sites of the four species are located at low elevations (250–330 m a.s.l.) in the Northeast of Austria. This region is characterized by high temperatures (8.9–9.6°C mean annual temperature, MAT), low precipitation (504 to 612 mm annual precipitation sum, APS), and long-lasting drought periods (e.g., Auer et al., 2005; Efthymiadis et al., 2006; Nobilis and Godina, 2006), conditions under which coniferous trees in Central Europe do not regularly occur (Table 1). In fact, the trial sites are located very close to the warm and dry distribution limit of *P. abies, A. alba*, and *L. decidua*, while, for *P. menziesii*, it is still within the warmest quarter of distribution (Figure 1). All four trials were established in the 1970s with 3- to 4-year-old seedlings in randomized block designs (see Table 1 for further details).

**Table 1:** Trial sites and basic climate descriptors of trial sites in Eastern Austria.

**Treespecies**	**Site**	**Latitude**	**Longitude**	**Altitude (m)**	**Establishment year**	**Plant spacing**	**MAT (°C)**	**APS (mm/year)**
Norway spruce	Porrau	48.548	16.171	250	1978	1.8 × 1.5 m	9.1	524
Douglas-fir	Traismauer	48.333	15.777	320	1976	2.0 × 2.0 m	8.9	580
Silver fir	Knödelhütte	48.218	16.241	300	1970	0.5 × 1.0 m	9.6	621
European larch	Zlabern	48.724	16.540	330	1978	2.0 × 2.0 m	9.4	504

*Climate data were calculated from station data according to Chakraborty et al. (2015). MAT, mean annual temperature (1971–2000); APS, annual precipitation sum (1971–2000)*.

Provenances of the three European species originate from habitats within a narrow precipitation range of approximately 600–1,400 mm per year, while provenance of Douglas-fir originates from sites receiving between 500 and 2,300-mm precipitation/year (Figure 1). Douglas fir provenance also originates from the coldest sites (MAT_min_ = 0.3°C), while provenance of silver fir originates from the warmest (MAT_max_ = 15.7°C).

For the analysis of the present study, 9–16 provenances per species were selected, representing the distribution of the species as wide as possible (Supplementary Figure 1). From each of the selected provenances, on average, 16 trees were sampled by taking two increment cores at breast height (Supplementary Table 1).

### Sample Processing

Tree cores were cut into cross sections of approximately 1.4 mm with a double-blade circular saw. These cross sections were placed on microfilms and exposed to a 10-kV (24 mA) X-ray source for 25 min. The obtained microfilms were analyzed with WinDENDRO 2009 (Regent Instrument, Quebec, CAN), and ring widths for each year were measured to the nearest 0.001 mm. In order to obtain the climatic and genetic variance and to reduce non-climatic noise (e.g., from reaction wood), values from the two cores of the same tree were averaged. Data were converted into single-tree time series and mean chronologies, using the dplr package in R (R Development Core Team, 2008; Bunn, 2010).

### Identification of Drought Events

Drought periods with consequences for tree growth within the last three decades were identified by the standardized precipitation index SPI according to McKee et al. (1993). The SPI relates the precipitation deficit or surplus during a certain time period to the mean and standard deviation of precipitation throughout the time series. Although the SPI is based on monthly precipitation time series (in our case, from 1970 to 2010), it can be used to identify drought on time scales of 1–48 months and thus allows to differentiate between frequently occurring short droughts and rare, long-enduring events. To account for droughts on all possible time scales, the software SPI SL 6 (NDMC, 2014) was applied to calculate the SPI for periods of 1, 3, 6, 12, 24, as well as 48 months. Climate data were obtained from the four nearest weather stations of the Austrian Central Station for Meteorology and Geodynamic (ZAMG) network and interpolated by inverse distance-weighted interpolation to the coordinates of the site (described in Chakraborty et al., 2015).

**Figure 2:**
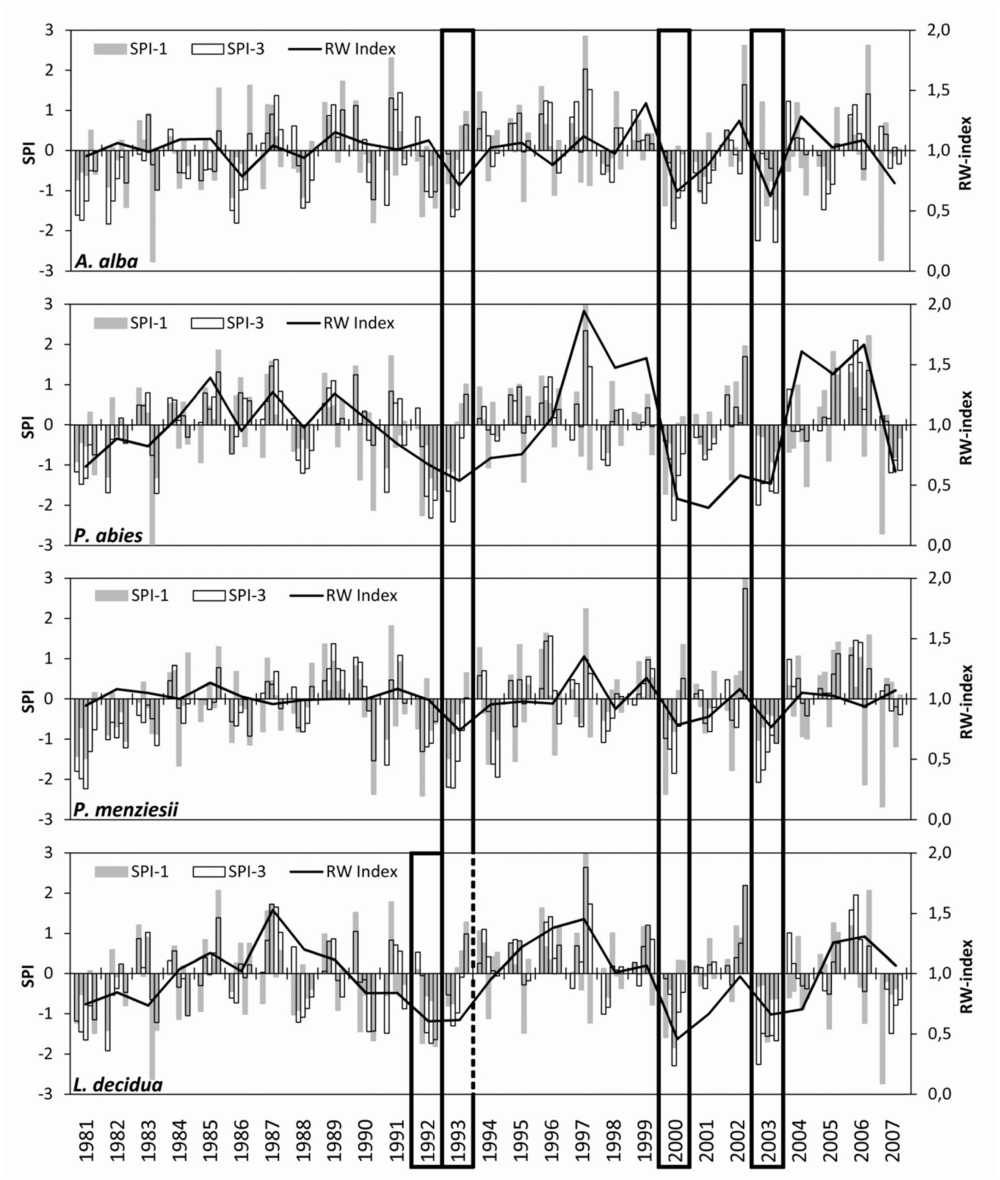
Drought occurrence and resulting increment declines on four conifer trial sites in the Northeast of Austria. The bars show the standardized precipitation index SPI (given in standard deviations) on time scales of 1 (SPI-1) and 3 (SPI-3) months. The line plots illustrate the course of an annual increment (as a dimensionless ring-width index). Frames mark the most significant and common drought periods that are used for comparison among trials and species.

All four trial sites experienced several moderate (SPI < −1), severe (SPI < −1.5), and extreme (SPI < −2) drought events between 1981 and 2007 (Figure 2). The most remarkable droughts occurred in the early 1990s, i.e., the years 1992 and 1993, in 2000, and in 2003, when SPI < −1.5 was observed at all sites *n* monthly and/or three-monthly time periods. These drought periods resulted in noticeable declines of increment for all species and were thus selected for comparative analysis. All sites experienced droughts in 1992 and 1993, but only at the trial site of European larch; drought in 1992 was more intense than in 1993, causing the greatest growth decline 1 year ahead of the other three species. Generally, the coincidence of drought events across sites was high as demonstrated by correlations between *R* = 0.75 and *R* = 0.94 among the SPI time series of the trials (Supplementary Table 2).

### Characterization of Drought Response

The performance of individual trees during drought events was evaluated by two measures of drought response according to Lloret et al. (2011): resistance and recovery. Resistance (Res) characterizes how much a tree is reducing its incremental growth during drought and is calculated by the ratio of the annual increment during drought (*I*_dr_) to the average growth performance across a time period of 9 years (*I*_9yr_), with the drought event in the central year by Res = *I*_dr_/*I*_9yr_. A 9-year average as reference radial growth was used to overcome year-to-year growth variation and the consecutive occurrence of drought within few years. Here, trees with a Res = 1 do not show any deviance from the 9-year mean and decreasing values of Res stand for increasing drought sensitivity. Recovery (Rec) is calculated by the ratio (Rec = *I*_postdr_/*I*_dr_) of the mean annual increment across the 2 years, following a drought year (*I*_postdr_), to the increment during a drought event (*I*_dr_), and describes how fast a tree is able to recreate after a certain drought period. Higher recovery stands for faster revitalization. The two drought-response measures were calculated from raw, untransformed ring-width series of each tree, because the biological age-related growth decrease can be neglected within the relatively short time frame of each individual drought event and the comparable age of the trees at trial sites (Pretzsch et al., 2013). Also, transformations into the widely used basal area increment were unsuitable as any change of dimensionality of the measured traits affects the trait variances and thus the estimates of variance components and genetic parameters (Houle, 1992).

### Statistical Analysis

#### Variation Among Species

To test for differences in resistance and recovery among species and among different drought events, a two-way ANOVA was used, where tree species and a drought year were treated as independent effects, allowing for interaction between species and drought.

#### Variation and Stability Among Provenances

In order to reveal differences among provenances and different drought years, we performed a repeated measure ANOVA for each single species. Here, single trees were treated as random effects and “provenance” and a “drought event” as fixed between-group variables according to Moser et al. (1990): *y*_*i*_ = μ + ε_*i*_, where *y*_*i*_ represents the ith-repeated measurement of drought response made on the same tree, μ the vector of means for each provenance/drought event combination, and ε_*i*_ the vector of random errors of measurements made on the same tree. This multivariate approach reduces potential biases caused by carry-over effects from previous drought events (Moser et al., 1990). Between-group variables and their interactions were included stepwise and compared with a baseline model without other predictors than the intercept. The Akaike information criterion (AIC) and the log likelihood and the likelihood ratio were used for model evaluation. These steps were performed in the nlme package in R (R Development Core Team, 2008) for linear mixed-effects models.

To quantify the stability of provenances across consecutive drought events, we determined the phenotypic stability of each individual provenance throughout the observed drought events according to Finlay and Wilkinson (1963). Therefore, the mean drought response (for Res and Rec) of all provenances within each single drought period was calculated to provide a baseline for drought response in relation to the respective drought intensity or duration, respectively. Then, the performance of a single provenance was linearly regressed to the mean of all provenances, and the regression coefficient *b* was estimated. Provenances characterized by regression coefficients around *b* ≈ 1 show average stability with low resistance if the mean of all provenances is poor, and high resistance if the mean response is high. Provenances with *b* > 1 show above-average resistance when the mean resistance is high, but below-average resistance when mean resistance is low. Given that lower average resistance can be found under more extreme drought events, such provenances, therefore, show low stability and increasing sensitivity to environmental stress. Provenances with *b* < 1 show above-average resistance if the mean of all provenances is poor (i.e., under stronger drought) and below-average resistance if mean resistance is high. Such provenances show above-average stability and high resistance to extreme drought events.

In order to compare stability across species, we calculated the absolute value of the provenance deviation from 1 for each provenance and averaged them up across species: $b_{dev}=\bar{\left|1-b_{n}\right|}$. Moreover, the average instability for each species was computed by $b_{ist}=\bar{b_{n}}$ for all provenances *n* with *b* > 1.

#### Degree of Genetic Determination and Evolvability of Provenances

The degree of genetic determination was estimated by the repeatability of the two drought-response measures, i.e., by the repeated occurrence of drought events at our trial site and individual reactions of the trees to these drought events. Repeatability *r* gives the proportion of total variation of a certain trait that is due to differences between individuals. It is related to the heritability in the narrow sense, as the additive genetic variance *V*_*A*_, the numerator of heritability, can never be larger than the numerator of the repeatability *V*_*G*_ + *V*_*EG*_ (Falconer and Mackay, 1996) within its theoretical formulation: *r* = $\frac{V_{G}+\;V_{EG}}{V_{P}}$. Here, *V*_*G*_ is the genotypic variance; *V*_*EG*_, the general environmental variance; and *V*_*p*_, the total phenotypic variance. Thus, repeatability *r* determines the upper limit of heritability of the given trait (Boake, 1989; Merilä and Sheldon, 2000, but see Dohm, 2002). The repeatability *r* can be calculated by the ratio of the among-group variance $\sigma_{A}^{2}$ to the total phenotypic variance $\sigma_{P}^{2}$, where $\sigma_{P}^{2}$ is the sum of among and within-group variance $\sigma_{W}^{2}$ according to Sokal and Rohlf (1995): $r=\frac{\sigma_{A}^{2}}{\left(\sigma_{A}^{2}+\sigma_{W}^{2}\right)}$. Both, the among-group variance $\sigma_{A}^{2}$ and the within-group variance $\sigma_{W}^{2}$ were estimated using restricted maximum-likelihood estimation (REML) in the software ASReml (Gilmour et al., 2009). For these calculations, drought-response indices were standardized and log-transformed to achieve a normal-like distribution (Nakagawa and Schielzeth, 2010).

By assuming equal selection pressures across all provenances of a species, the overall repeatability of the respective species was estimated, using a mixed-effect model with individual trees and provenances as random effects. Moreover, we calculated provenance-specific repeatability in univariate mixed-effect models with individual trees as a single random effect. These later models allow for comparing repeatability among provenances under the assumption that the environmental origin of the provenances resulted in different selection pressures on drought response. Repeatabilities and their standard errors were calculated with the postprocessing module of ASReml (Gilmour et al., 2009).

Another meaningful measure of the quantitative genetic variation within populations is evolvability, an indicator for its ability to respond to natural or sexual selection (Houle, 1992; Garcia-Gonzales et al., 2012). A relevant measure for evolvability is the dimensionless coefficient of additive genetic variation *CV*_*A*_, which—because it is standardized by the trait mean—allows comparing the evolvability of traits, species, and populations. In our case, repeatability r corresponds to the upper limit of heritability $h_{\max}^{2}$, within a provenance and is used to calculate the potential additive genetic variation $V_{A}=h_{\max}^{2}\;*V_{P}$. The coefficient of additive genetic variation—now, the upper limit of evolvability is then calculated by *CV*_*A*_ = $100*\sqrt{\frac{V_{A}}{\bar{X}}}$ (Houle, 1992), with $\bar{X}$ being the provenance mean of resistance and recovery, respectively.

## Results

### Variation Among Species

**Figure 3:**
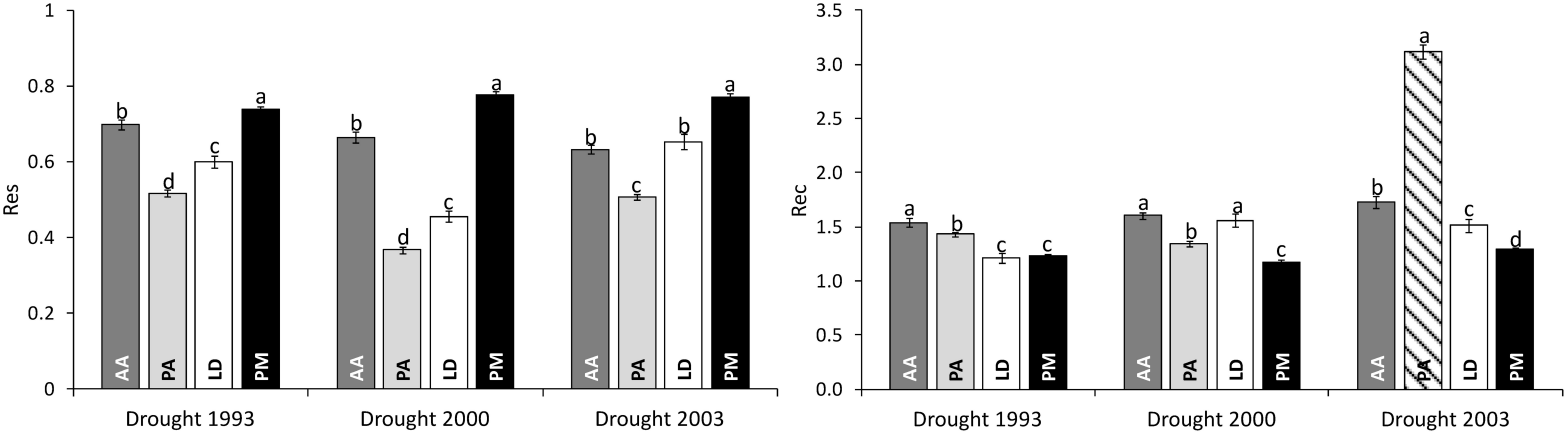
Comparison of drought response of four conifers across three severe drought periods as given by Resistance (Res) and Recovery (Rec). Bar plots represent the mean values of all individuals of a certain species; whiskers give the standard error. The striped bar marks a biased estimate of recovery due to the short time interval of subsequent drought events. AA, *Abies alba*; PA, *Picea abies*; LD, *Larix decidua*; PM, *Pseudotsuga menziesii*. Lower case letters a, b, c, and d mark significant differences determined in pairwise *post-hoc* tests.

Significant differences in drought response were found among species as well as among drought periods for both response measures (Table 2). Across all drought periods, Douglas-firs revealed the highest resistance followed by silver fir, European larch, and Norway spruce (Figure 3). After a drought, silver fir was found to exhibit the highest recovery and Douglas-fir the worst. Due to the strong growth decline of Norway spruce during drought 2000 (see also Figure 2), the recovery of this species after 2003 (striped bar in Figure 3) is biased and likely overestimates the species potential as recovery after 1993 and 2000 was much lower.

**Table 2:** ANOVA for comparison of drought response among tree species and drought years.

**Response variable and factor**		**Resistance**		**Recovery**	
	***dF***	***F***	***p***	***F***	***p***
Species	3	479.64	<0.005	178.29	<0.005
Drought year	2	49.41	<0.005	151.69	<0.005
Species × Drought year	6	27.30	<0.005	139.78	<0.005

### Variation and Stability Among Provenances

**Figure 4:**
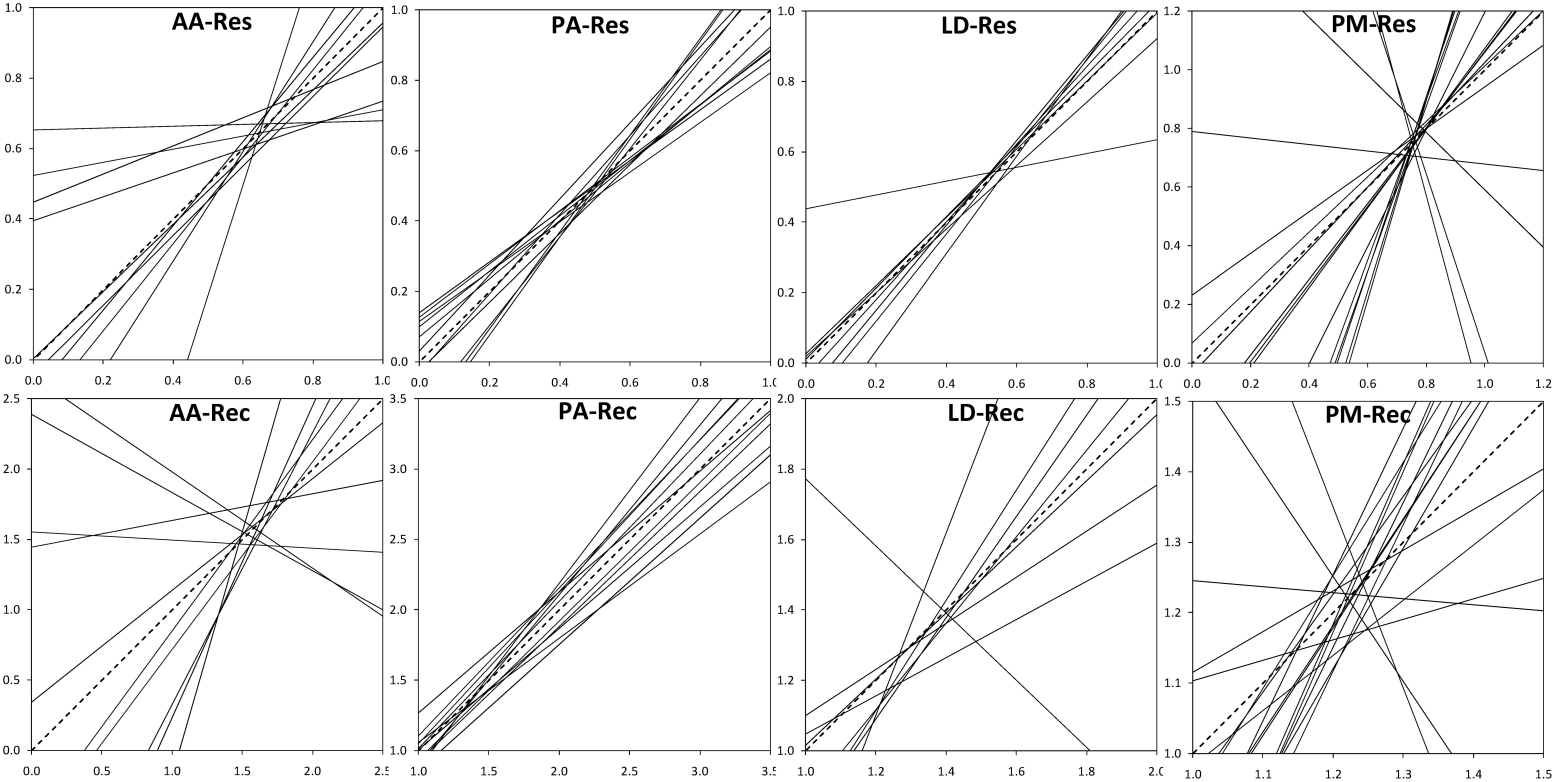
Phenotypic stability of provenances throughout the three drought events according to Finlay and Wilkinson (1963). Dashed lines indicate the average stability. Provenances with high stability show slopes (regression coefficients) <1, whereas slopes >1 stand for instable provenances. Upper row: Resistance, Res; lower row: recovery, Rec. AA, *Abies alba*; PA, *Picea abies*; LD, *Larix decidua*; PM, *Pseudotsuga menziesii*.

The analysis of the intraspecific variation of drought response confirmed the significant effect of drought events, as it was found to be a significant factor in the repeated measure ANOVA for all four species (Table 3). In addition, significant provenance variation was found for resistance and recovery in Norway spruce and silver fir. Differences among provenances of European larch and Douglas fir were smaller and neither significant for resistance nor for recovery. However, for all four species, we found significant interactions between drought events and provenances, indicating that rank shifts occur among provenances (Table 3). This is also supported by the huge variation in phenotypic stability found among provenances and species (Table 4, Figure 4). Highest stability was found for provenances of Norway spruce, where all provenances exhibit moderate values of Finlay and Wilkinson (1963) *b*, ranging from stable (*b* = 0.7) to slightly unstable (*b* = 1.4) and resulting into an average deviation from the population mean of *b*_*dev*_ = 0.22 for Res and 0.13 for Rec. European larch exhibits similar stability across provenances for Res (*b*_*dev*_ = 0.23), but not for Rec (*b*_*dev*_ = 0.70) where *b* ranges from −1 to 2.6 (Figure 4). In contrast, provenances of Douglas fir and, to a lesser degree, of silver fir did show markedly lower stability across drought periods (Table 4, Figure 4).

**Table 3:** Variation among provenances and drought years for individual species as revealed by repeated measure ANOVA.

				**Resistance**				**Recovery**			
		**Model**	***df***	***AIC***	**logLik**	**L. Ratio**	***p*-Value**	**AIC**	**logLik**	**L. Ratio**	***p*-Value**
*A.alba*	Intercept	1	3	−342.13	174.07			527.29	−260.65		
	Drought	2	5	−349.30	179.65	11.17	**0.0038**	523.26	−256.63	8.04	**0.0180**
	Provenance	3	15	−349.85	189.92	20.55	**0.0245**	513.44	−241.72	29.82	**0.0009**
	Drought × Provenance	4	35	−342.64	206.32	32.79	**0.0356**	515.66	−222.83	37.78	**0.0094**
*P. abies*	Intercept	1	3	−792.10	399.06			3,008.26	−1,501.13		
	Drought	2	5	−1,008.26	509.13	220.16	**<0.0001**	2,303.09	−1,146.55	709.17	**<0.0001**
	Provenance	3	15	−1,011.44	520.72	23.18	**0.0101**	2,295.41	−1,132.70	27.68	**0.0020**
	Drought × Provenance	4	35	−1,023.10	546.55	51.66	**0.0001**	2,298.10	−1,114.05	37.31	**0.0108**
*L.decidua*	Intercept	1	3	−135.95	70.98			676.30	−335.15		
	Drought	2	5	−203.21	106.60	71.26	**<0.0001**	655.82	−322.91	24.47	**<0.0001**
	Provenance	3	12	−193.10	108.55	3.89	0.7920	659.81	−317.90	10.02	0.1877
	Drought × Provenance	4	26	−207.06	129.53	41.96	**0.0001**	648.66	−298.33	39.15	**0.0003**
*P. menziesii*	Intercept	1	3	−555.31	280.66			41.42	−17.71		
	Drought	2	5	−558.28	284.14	6.97	**0.0307**	25.77	−7.88	19.65	**0.0001**
	Provenance	3	20	−550.97	295.48	22.69	0.0910	38.06	0.97	17.71	0.2782
	Drought × Provenance	4	50	−598.42	349.21	107.45	**<0.0001**	35.81	32.09	62.25	**0.0005**

*Bold numbers show significant effects*.

**Table 4:** Average phenotypic stability of provenances throughout the observed drought events as revealed by Spearman's rank correlations *R*_*s*_ and Finlay and Wilkinson (1963) regression, where *b*_*dev*_ is the mean deviation of all provenances from the species mean and *b*_*ist*_, the mean deviation of all instable provenances from the species mean.

	**Resistance**		**Recovery**	
	***b_***dev***_***	***b_***ist***_***	***b_***dev***_***	***b_***ist***_***
*A. alba*	0.62	0.31	1.06	0.53
*P. abies*	0.22	0.11	0.13	0.06
*L. decidua*	0.23	0.12	0.70	0.35
*P. menziesii*	1.53	0.76	1.06	0.53

### Degree of Genetic Determination and Evolvability of Provenances

Differences in the degree of genetic determination of drought response were found among species and among provenances. Calculating repeatability across provenances revealed significant repeatabilities between 0.11 and 0.18 only for Norway spruce and European larch (Table 5). When we supposed varying selection regimes in the provenance origin and applied univariate mixed-effect models for each individual provenance, we observed high variation in repeatabilities among provenances (Table 5, Supplementary Tables 3–6). Within every species, some provenances did not show significant repeatability. For resistance, the highest number of provenances (55%) with significant repeatabilities up to 0.44 was found in Norway spruce. In contrast, none of the Douglas-fir provenances did reveal a significant degree of genetic determination.

**Table 5:** Repeatability of resistance and recovery as estimated by the repeatability of drought response across drought events.

	**Resistance**				**Recovery**			
	***r*_**sp**_**	***n*_**p**_**	***r*_**prange**_**	***r*_**pmean**_**	***r*_**sp**_**	***n*_**p**_**	***r*_**prange**_**	***r*_**pmean**_**
*A. alba*	0.037 ± 0.055	2 of 10 (20%)	0–0.468	0.357	0.012 ± 0.052	2 of 10 (20%)	0–0.527	0.4415
*P. abies*	**0.129** **±** **0.035**	6 of 11 (55%)	0–0.440	0.335	**0.182** **±** **0.036**	5 of 11 (45%)	0–0.324	0.247
*L. decidua*	**0.112** **±** **0.059**	3 of 8 (38%)	0–0.319	0.271	**0.161** **±** **0.061**	5 of 8 (63%)	0–0.425	0.3118
*P. menziesii*	0.000 ± 0.000	0 of 16 (0%)	–	–	0.000 ± 0.000	2 of 16 (13%)	0–0.236	0.221

*r_sp_, repeatability for species, tested across all provenances by taking provenances into account as random effect. r_pmean_, mean repeatability of provenances for which significant provenance-specific repeatability was found. r_prange_, observed range of provenance-specific repeatabilities. n_p_, number (and frequency) of provenances with significant repeatabilities among all analyzed provenances of a species. Bold numbers show significant repeatabilities*.

For recovery, the highest number of provenances (63%) with significant repeatabilities up to 0.43 was found in European larch. Also, 2 out of 16 provenances of Douglas-fir and 2 of 10 provenances of silver fir revealed significant repeatabilities. The highest repeatabilities for a single provenance were found in silver fir (Supplementary Tables 3–6).

**Figure 5:**
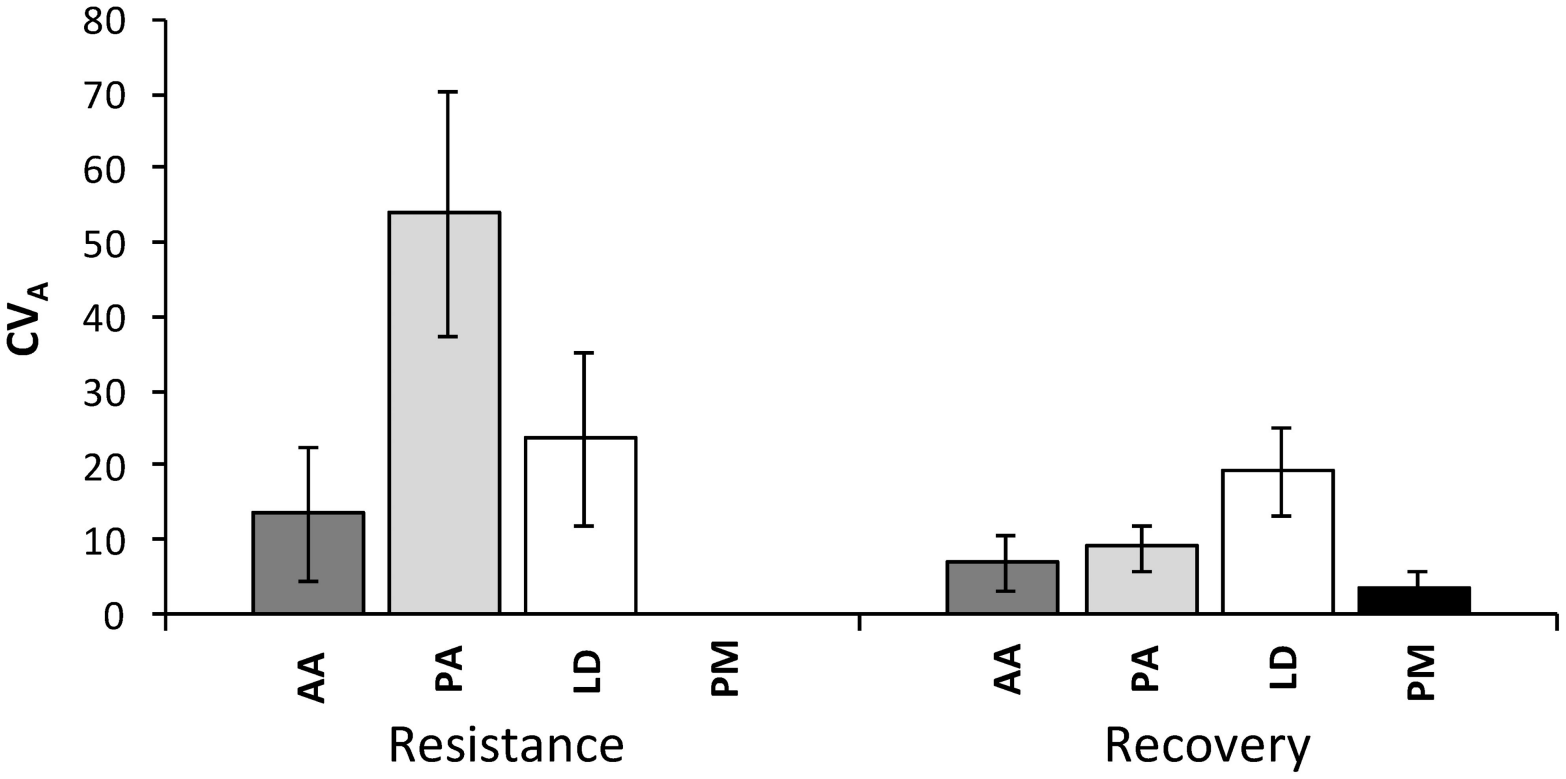
Evolvability of drought response (resistance and recovery) within provenances given as an additive genetic coefficient of variation CV_A_ across all provenances of each species. Whiskers mark the standard error of individual provenances. AA, *Abies alba*; PA, *Picea abies*; LD, *Larix decidua*; PM, *Pseudotsuga menziesii*.

**Figure 6:**
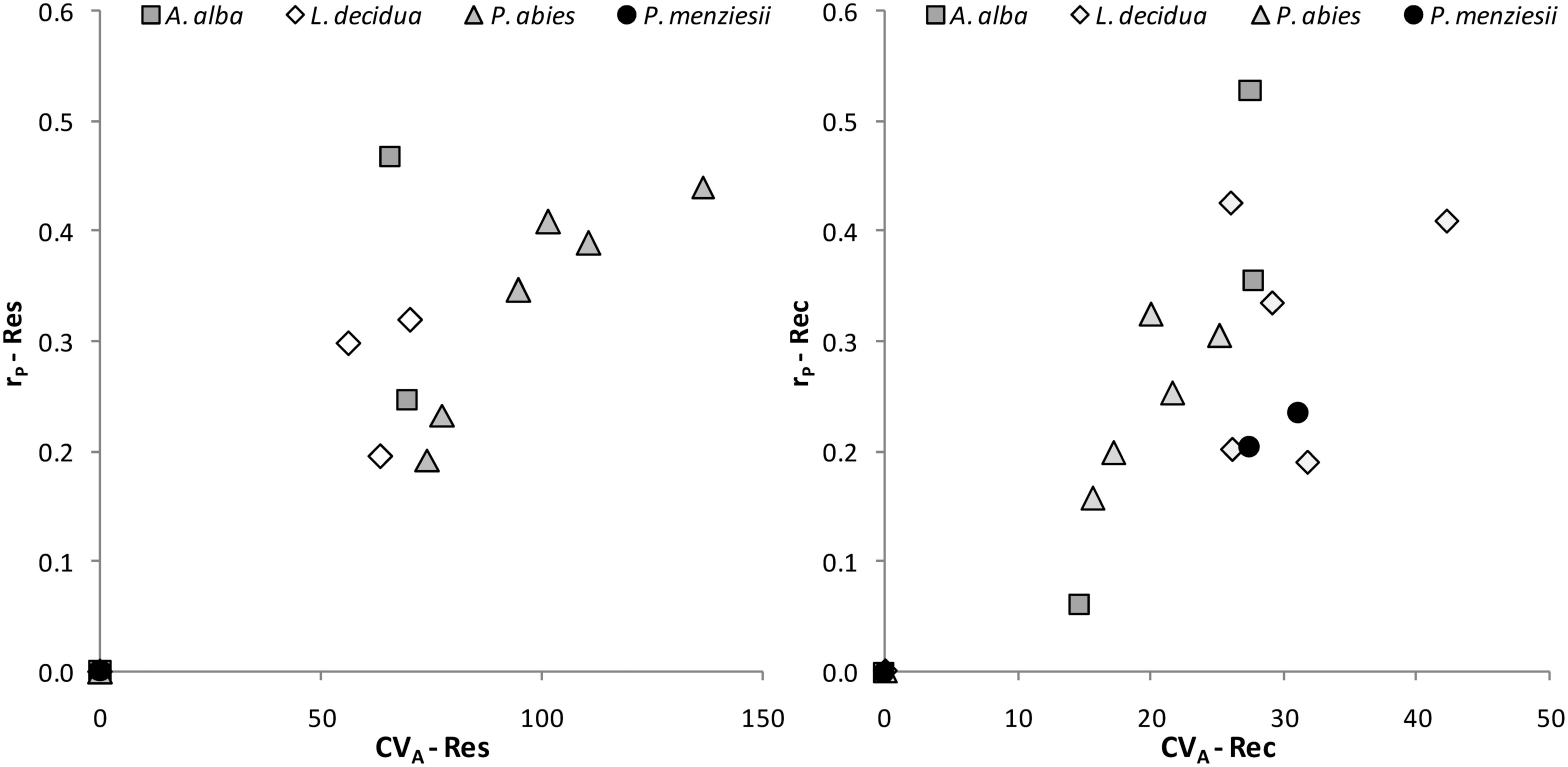
Evolvability of drought response of individual provenances as given by the coefficient of additive genetic variation *CV*_*A*_ and the degree of genetic determination, i.e., the repeatability r_P_ for resistance (Res) and recovery (Rec).

The coefficient of additive genetic variation *CV*_*A*_ confirmed the lower evolvability of Douglas-fir populations in terms of drought resistance, but not of drought recovery (Figure 5). Generally, both the degree of genetic determination and *CV*_*A*_ were higher across provenances of spruce and larch and lowest, respectively, zero for provenances of Douglas-fir (Figure 6).

## Discussion

Intraspecific variation in drought response of forest trees is a key determinant for understanding and managing the fate of forest ecosystems in climate change. Our analysis strongly suggests that the general vulnerability of a tree species to drought might not necessarily be connected to intraspecific genetic variation and evolvability in drought response.

### Interspecific Variation in Drought Response

The general ability of species to cope with drought revealed the highest resistance for Douglas-fir and the lowest resistance for Norway spruce. Drought recovery was highest for a silver fir, followed by larch and Norway spruce. This is in good agreement with similar studies across Europe. A comparison of drought response in Austrian pine (*P. nigra*), Scots pine (*P. sylvestris*), Douglas-fir, and European larch in Switzerland revealed the highest sensitivity for larch, while Douglas fir showed only modest growth reductions and better recovery (Eilmann and Rigling, 2012). More comprehensive Swiss studies (Lévesque et al., 2013, 2014) confirmed that, besides larch, also Norway spruce and Scots pine are highly drought sensitive, especially when growing close to their xeric distribution limit. Also, Zang et al. (2014) and Vitali et al. (2017) confirmed the weak performance of Norway spruce, which showed the lowest resistance and resilience as compared with silver fir and Douglas-fir.

### Genetic Variation in Drought Response Among Provenances

The good agreement of our among-species comparison to other European studies supports the suitability of our provenance trial network to infer also the genetic variation among and within the tested provenances. However, our provenance sampling was biased by the availability of populations within the existing trials, which do not necessarily encompass the full representativity of the species, subspecies, or local adaptations within the species. We found significant variation in drought resistance and recovery among provenances of silver fir and Norway spruce, but not among provenances of European larch and Douglas fir. Although there is, so far, no study comparing among-provenance variation across species, a handful of single species studies confirm the observed differences. Most comprehensive among-provenance data are, so far, available for Douglas-fir, providing ambiguous findings of its genetic variation in drought response. On the one hand, trade-offs between productivity and drought tolerance have been found when comparing provenances growing within trials located in western Europe (Eilmann et al., 2013) and western North America (Montwe et al., 2015). On the other hand, statistical support for differences among provenances in drought response of early and latewood increment (Eilmann et al., 2013) and resistance of diameter growth (Montwe et al., 2015) is weak in both experiments, confirming the low among-provenance variation found in our continental test site. However, while the immediate radial increment during a drought might not differ among Douglas-fir provenances, studies on other traits such as wood density suggest that wood density profiles or intra-annual density fluctuations could be more valuable traits for selecting drought-resistant provenances and individuals (Martinez-Meier et al., 2008a,b; George et al., 2019).

In contrast to Douglas-fir, comparable studies in European conifers are rare. For European larch, only one single study tested for differences in drought response so far (George et al., 2017) and detected significant differences among provenances within a 50-year-old experiment. This trial contained international provenances across huge parts of the natural larch distribution, whereas the geographic coverage of seed sources in the present study is smaller, representing only the eastern Alpine range of the species. Thus, it seems likely that provenances of this area are not sufficiently differentiated, because George et al. (2017) observed that, particularly, provenances from the Polish lowlands—not represented here—showed the best drought performance.

Also, for Norway spruce, the economically most important and most widespread conifer of Central and Northern Europe, the genetic variation in drought sensitivity among provenances, has rarely been addressed. An early study on 17 Polish provenances (Burczyk and Giertych, 1991) did not detect differences, probably because the provenances originate from a limited geographic region where only one phylogeographic lineage occurred. In the current study, genetic material from a broader geographic range has been tested, including provenances of the western Carpathian Mountains, the Bohemian massif, the eastern Alpine range, and the Rhodope Mountains (Trujillo-Moya et al., 2018). This region harbors high genetic diversity due to multiple refugial areas and postglacial migration history (Tollefsrud et al., 2008; Mihai et al., 2020). Besides testing for immediate effects of extreme drought on radial growth, further studies on Norway spruce found significant provenance variation of the long-term growth reactions to climate parameters (i.e., Suvanto et al., 2016; Klisz et al., 2019) or different physiological strategies of seedlings to cope with water limitations (Jamnická et al., 2019).

Evidence for genetic variation of climate-growth response within silver fir comes from dendroecological analysis throughout the Carpathian Mountains, demonstrating that radial growth within populations originating from Apennine origin, is more strongly driven by summer temperatures, whereas the increment of Balkan populations is more driven by summer drought (Bosela et al., 2016). Generally, genetic variation in the growth of silver fir seems to be driven by both postglacial recolonization and natural selection (Martínez-Sancho et al., 2021). Also, a more comprehensive analysis of the present silver fir trial across six drought periods between 1986 and 2007 revealed significant differences among provenances within three of these events (George et al., 2015) but also found frequent rank changes in the drought performance of individual provenances. In our comparison, silver fir provenances were found to have relatively low phenotypic stability across consecutive drought periods when compared with European larch and Norway spruce (Table 3). Thus, superior provenances within one drought period were not necessarily performing well during another period, a behavior that has been connected to the seasonal appearance of the respective drought event and possible interactions with phenological changes as, for example, bud burst, the start of radial growth or the growth transition from early to latewood (George et al., 2015, 2019). In contrast, provenances of Norway spruce and larch seem to be rather insensitive to the seasonality of drought occurrence and show comparable genetic variation across all events, thus facilitating the selection of drought-adapted provenances for assisted seed transfer.

### Genetic Variation in Drought Response Among Individuals

Besides among-provenance variation, we also found significant genetic variation among individuals within some but not all of the tested provenances. This strongly indicates that selection regimes in response to drought events likely vary among the provenance origins of the four tested conifers. The evolutionary background of this provenance variation is likely related to the varying climatic origin of the tested provenances (Figure 1) but may also relate to their phylogeographic origin from different refugial lineages, which are often shaped by divergent selection (Chen et al., 2012, 2016).

The observed repeatabilities for different provenances range from 0.157 (recovery of provenance Q14, Norway spruce) up to 0.527 (recovery of provenance 90, silver fir). This order of magnitude resembles estimates of broad-sense heritability of hydraulic conductivity for Norway spruce, which ranged from 0.14 to 0.31 for different conductivity parameters (Rosner et al., 2007, 2008). Also, wood density, which was found to be a strong indicator for drought response (Rosner et al., 2014), shows heritabilities in the range of 0.10 and 0.41 for Norway spruce (Rozenberg et al., 2002) or mean values around 0.5 for conifers in general (Cornelius, 1994). Physiological measures related to drought response were found to be on a similar medium level as shown in our comparison, for example, the water use efficiency of trees as estimated by carbon isotope discrimination δ13 revealed heritabilities between 0.09 for *Pinus taeda* (Baltunis et al., 2008), 0.23–0.41 for *Pinus pinaster* (Marguerit et al., 2014), and 0.54 for *Picea mariana* (Johnsen et al., 1999).

Interestingly, Douglas-fir and silver fir, which showed the highest mean resistance and mean recovery, respectively, showed the lowest genetic variation among individuals within provenances. In Douglas-fir, only two of the 16 Douglas fir provenances exhibited significant repeatability in recovery and none for resistance, and, in silver fir, 2 of 10 provenances showed significant repeatability for resistance and recovery. In contrast, five and six provenances of Norway spruce, as well as three and five provenances of larch showed significant repeatability for resistance and recovery, respectively. These contradictory findings for Douglas-fir and silver fir could potentially be explained by too little drought intensity, which might have been too weak to provoke a genetically driven individual drought response. Zang et al. (2014) observed comparable drought-response patterns in different tree species, showing that less favorable site conditions increased the variability in drought response among individuals within populations. In Douglas-fir, a stronger expression of drought resistance was also observed at experiments within warm and dry environments due to genotype x environmental interactions (Bansal et al., 2015). Thus, extreme drought conditions close to the species-specific limits might be required to identify genetic variation.

Another explanation for the low variation within silver fir populations could be their status as shade-tolerant climax species, which are most successful within late successional stages of forest development. These stages are characterized by relatively good soil conditions and forest regeneration within the shade of other species. Thus, within its early life stages, in which intraspecific competition and environmental selection within populations are most distinct (Kapeller et al., 2017), silver fir is rarely exposed to direct radiation, extreme heat, and a low water status. And, hence, we cannot expect that seeds collected from such populations may contain strong within-population variation. In contrast, European larch is a light-demanding pioneer species, in particular when growing within subalpine environments where shallow soils and high-solar radiation in the summer months may lead to regular water shortages. Such environments are expected to favor natural selection and genetic variation in drought response within forest sites and populations. Norway spruce and Douglas-fir take intermediate positions as both grow with a pioneer character in subalpine environments in potentially drought-prone habitats, while they also grow as climax species within late successional stages. Thus, we should expect to find populations with high- and low-genetic variation in drought response. The distinction between these species could be the widespread plantation of Norway spruce in clear-cut systems throughout the last 200 years across Central Europe. Within such open reforestation, trees more likely to face environmental selection (Kapeller et al., 2017). In contrast, the tested Douglas-fir provenances originate from primary old-growth forests (Kleinschmit and Bastien, 1992), where putatively environmental selection during juvenile growth was rather limited. Unfortunately, exact information on the history and local site conditions of the seed source populations is mostly not available.

Direct comparisons of genetic repeatability values with estimates of heritability should consider that repeatability represents the upper limit of heritability, and, thus, true heritability could be lower, but never larger than repeatability. The same is true for the estimated coefficient of additive genetic variation, which gives—because it is based on repeatability—rather the upper limit of evolvability. Evolvability, the ability of a population to respond to natural selection, shall not be confined to heritability, which might be uncorrelated to evolvability and is a poor predictor of response to natural selection (Hansen et al., 2011). In our analysis, repeatability, and the coefficient of additive genetic variation provide a comparable ranking across provenances and species. Future studies should aim at confirming the quantitative variation by means of molecular studies testing for traditional sources of variation, such as DNA mutation and recombination, as well as for nongenetic mechanisms as, for example, stochastic gene expression or epigenetic modifications (Payne and Wagner, 2019).

## Conclusions and Applications

The most discussed measures to adapt European forests to climate change and to withstand more frequent droughts encompass (i) the replacement of conifers with native deciduous tree species and species mixtures (Jandl et al., 2015), as well as (ii) the extended cultivation of non-native trees (Chakraborty et al., 2015, 2016). However, both measures were also discussed to have significant impacts on the provision of ecosystem services, in particular in terms of wood production (Hanewinkel et al., 2012) and the conservation of native forest biodiversity (Brundu and Richardson, 2016). Our study provides evidence that even highly drought-sensitive European conifers, such as Norway spruce and European larch, provide high-genetic variation among and within populations in drought response. Given the high vulnerability of genetic resources in climate change (Schueler et al., 2014), this genetic variation strongly needs to be conserved and utilized within active and passive forest adaptation strategies. Variation among populations should be utilized by transferring a less-drought-sensitive population in assisted gene-flow schemes across the natural distribution of species. Given the limited number of provenances per species and the exclusive focus on drought sensitivity traits, we cannot give specific assisted gene-flow guidance yet. This would require considering multiple traits, including growth, mortality, drought, and frost performance, for example. Our study also confirms that single populations of threatened conifers may contain significant genetic variation in drought response within forest stands. Although our study, which was restricted to provenances selected and planted more than 40 years ago, did not allow a representative comparison of provenances across the full-species ranges; the observed differences in drought response among and within provenances provide strong arguments for the utilization of such genetic variation for forest adaptation measures. This knowledge should be applied in two ways: first, conifer breeding programs need to incorporate genetic variation in drought response as a regular screening trait by installing greenhouse experiments and field trials in specifically drought prone environments. Secondly, passive adaptation should be fostered in existing conifer populations suffering ongoing and future drought events. Such stands might not completely be removed and replaced by other tree species, as they might contain significant evolvability to adapt to the new conditions.

## Data Availability Statement

The raw data supporting the conclusions of this article will be made available by the authors, without undue reservation.

## Author Contributions

SS and MG designed the research. J-PG, MG, SK-A, KM, and RK critically reviewed the manuscript. SS wrote the manuscript. SS and J-PG analyzed the data. KM and SK-A conducted laboratory work and pre-processed the data. SS, MG, SK-A, KM, and RK conducted field work. All authors contributed to the article and approved the submitted version.

## Conflict of Interest

The authors declare that the research was conducted in the absence of any commercial or financial relationships that could be construed as a potential conflict of interest.

## References

[B1] Aitken S. N. Bemmels J. B. (2016). Time to get moving: assisted gene flow of forest trees. Evol. Appl. 9, 271–290. 10.1111/eva.12293 27087852 PMC4780373

[B2] Aitken S. N. Hannerz M. (2001). Genecology and gene resource management strategies for conifer cold hardiness, in Conifer Cold Hardiness, eds Bigras F. J. Columbo S. J. (Dordrecht: Kluwer Academic Publishers), 23–53. 10.1007/978-94-015-9650-3_2

[B3] Allen C. D. Macalady A. K. Chenchouni H. Bachelet D. McDowell N. Vennetier M.. (2010). A global overview of drought and heat-induced tree mortality reveals emerging climate change risks for forests. For. Ecol. Manage. 259, 660–684. 10.1016/j.foreco.2009.09.001

[B4] Aranda I. Cano F. J. Gascó A. Cochard H. Nardini A. Mancha J. A.. (2015). Variation in photosynthetic performance and hydraulic architecture across European beech (*Fagus sylvatica* L.) populations supports the case for local adaptation to water stress. Tree Physiol. 35, 34–46. 10.1093/treephys/tpu101 25536961

[B5] Auer I. Böhm R. Jurkovic A. Orlik A. Potzmann R. Schöner W.. (2005). A new instrumental precipitation dataset in the greater alpine region for the period 1800-2002. Int. J. Climatol. 25, 139–166. 10.1002/joc.1135

[B6] Baltunis B. S. Martin T. A. Huber D. A. Davis J. M. (2008). Inheritance of foliar stable carbon isotope discrimination and third-year height in *Pinus taeda* clones on contrasting sites in Florida and Georgia. Genet. Genomes 4, 797–807. 10.1007/s11295-008-0152-2

[B7] Bansal S. Harrington C. A. Gould P. J. St. Clair J. B. (2015). Climate-related genetic variation in drought-resistance of Douglas-fir (*Pseudotsuga menziesii*). Glob. Chang. Biol. 21, 947–958. 10.1111/gcb.12719 25156589

[B8] Barrett R. D. Schluter D. (2008). Adaptation from standing genetic variation. Trends Ecol. Evol. 23, 38–44. 10.1016/j.tree.2007.09.008 18006185

[B9] Bennett A. C. McDowell N. G. Allen C. D. Anderson-Teixeira K. J. (2015). Larger trees suffer most during drought in forests worldwide. Nat. Plants 28:15139. 10.1038/nplants.2015.139 27251391

[B10] Berlin M. Persson T. Jansson G. Haapanen M. Ruotsalainen S. Bärring L.. (2016). Scots pine transfer effect models for growth and survival in Sweden and Finland. Sil. Fenn. 50:1562. 10.14214/sf.1562

[B11] Boake C. R. B. (1989). Repeatability: its role in evolutionary studies of mating behavior. Evol. Ecol. 3, 173–182. 10.1007/BF02270919

[B12] Bosela M. Popa I. Gömöry D. Longauer R. Tobin B. Kyncl J.. (2016). Effects of postglacial phylogeny and genetic diversity on the growth variability and climate sensitivity of European silver fir. J. Ecol. 104, 716–724. 10.1111/1365-2745.12561

[B13] Bradford J. B. Bell D. M. (2017). A window of opportunity for climate-change adaptation: easing tree mortality by reducing forest basal area. Front. Ecol. Environ. 15, 11–17. 10.1002/fee.1445

[B14] Breshears D. D. Allen C. D. (2002). The importance of rapid, disturbance-induced losses in carbon management and sequestration. Glob. Ecol. Biogeogr. Lett. 11, 1–15. 10.1046/j.1466-822X.2002.00274.x

[B15] Broadmeadow M. S. J. Ray D. Samuel C. J. A. (2005). Climate change and the future for broadleaved tree species in Britain. Forestry 78, 145–161. 10.1093/forestry/cpi014

[B16] Brundu G. Richardson D. M. (2016). Planted forests and invasive alien trees in Europe: a code for managing existing and future plantings to mitigate the risk of negative impacts frominvasions. NeoBiota 30, 5–47. 10.3897/neobiota.30.7015

[B17] Bunn A. G. (2010). Statistical and visual crossdating in R using the dplR library. Dendrochronologia 28, 251–258. 10.1016/j.dendro.2009.12.001

[B18] Burczyk J. Giertych M. (1991). Response of Norway Spruce (*Picea abies* [L] Karst) annual increments to drought for various provenances and locations. Sil. Genet. 40, 146–152.

[B19] Cailleret M. Dakos V. Jansen S. Robert E. M. Aakala T. Amoroso M. M.. (2019). Early-warning signals of individual tree mortality based on annual radial growth. Front. Plant Sci. 9:1964. 10.3389/fpls.2018.01964 30713543 PMC6346433

[B20] Camarero J. J. Gazol A. Sangüesa-Barreda G. Oliva J. Vicente-Serrano S. M. (2015). To die or not to die: early warnings of tree dieback in response to a severe drought. J. Ecol. 103, 44–57. 10.1111/1365-2745.12295

[B21] Cech T. Tomiczek C. (1996). Zum Kiefernsterben in Niederösterreich. Forstschutz aktuell 17/18, 12–13.

[B22] Chakraborty D. Wang T. Andre K. Konnert M. Lexer J. M. Matulla C.. (2016). Adapting Douglas-fir forestry in Central Europe: evaluation, application, and uncertainty analysis of a genetically based model. Eur. J. For. Res. 135, 919–936. 10.1007/s10342-016-0984-5

[B23] Chakraborty D. Wang T. Andre K. Konnert M. Lexer M. J. Matulla C.. (2015). Selecting populations for non-analogous climate conditions using universal response functions: the case of Douglas-fir in Central Europe. PLoS ONE 10:e0136357. 10.1371/journal.pone.0136357 26288363 PMC4564280

[B24] Chen J. Källman T. Ma X. Gyllenstrand N. Zaina G. Morgante M.. (2012). Disentangling the roles of history and local selection in shaping clinal variation of allele frequencies and gene expression in Norway spruce (*Picea abies*). Genetics 191, 865–881. 10.1534/genetics.112.140749 22542968 PMC3389980

[B25] Chen J. Källman T. Ma X. Zaina G. Morgante M. Lascoux M. (2016). Identifying genetic signatures of natural selection using pooled population sequencing in *Picea abies*. G3 (Bethesda). 6, 1979–1989. 10.1534/g3.116.028753 27172202 PMC4938651

[B26] Chevin L. M. Collins S. Lefèvre F. (2013). Phenotypic plasticity and evolutionary demographic responses to climate change: taking theory out to the field. Funct. Ecol. 27, 966–979. 10.1111/j.1365-2435.2012.02043.x

[B27] Choat B. Jansen S. Brodribb T. J. Cochard H. Delzon S. Bhaskar R.. (2012). Global convergence in the vulnerability of forests to drought. Nature 491, 752–755. 10.1038/nature11688 23172141

[B28] Cornelius J. (1994). Heritabilities and additive genetic coefficients of variation in forest trees. Canad. J. For. Res. 24, 372–379. 10.1139/x94-050

[B29] Dai A. (2014). Increasing drought under global warming in observations and models. Nat. Clim. Chang. 3, 52–58. 10.1038/nclimate1633

[B30] DeSoto L. Caillleret M. Sterck F. Jansen S. Kramer K. Robert E. M. R.. (2020). Low growth resilience to drought is related to future mortality risk in trees. Nat. Commun. 11:545. 10.1038/s41467-020-14300-5 31992718 PMC6987235

[B31] Dohm M. R. (2002). Repeatability estimates do not always set an upper limit to heritability. Funct. Ecol. 16, 273–280. 10.1046/j.1365-2435.2002.00621.x

[B32] Efthymiadis D. Jones P. D. Briffa K. R. Auer I. Böhm R. Schöner W.. (2006). Construction of a 10-min-gridded precipitation data set for the Greater Alpine Region for 1800-2003. J. Geophys. Res. 111:D01105. 10.1029/2005JD006120

[B33] Eilmann B. de Vries S. M. G. den Ouden J. Mohren G. M. J. Sauren P. Sass-Klaassen U. (2013). Origin matters! Difference in drought tolerance and productivity of coastal Douglas-fir (Pseudotsuga menziesii (Mirb.)) provenances. For. Ecol. Manage. 302, 133–143. 10.1016/j.foreco.2013.03.031

[B34] Eilmann B. Rigling A. (2012). Tree-growth analyses to estimate tree species' drought tolerance. Tree Physiol. 32, 178–187. 10.1093/treephys/tps004 22363071

[B35] Fady B. Cottrell J. Ackzell L. Alía R. Muys B. Prada A.. (2016). Forests and global change: what can genetics contribute to the major forest management and policy challenges of the twenty-first century? Reg. Environ. Change 16, 927–939. 10.1007/s10113-015-0843-9

[B36] Falconer D. S. Mackay T. F. C. (1996). Introduction to Quantitative Genetics. London: Longmans Green.

[B37] Finlay K. W. Wilkinson G. N. (1963). The analysis of adaptation in a plant-breeding programme. Aust. J. Agric. Res. 14, 742–754. 10.1071/AR9630742

[B38] Garcia-Gonzales F. Simmons L. W. Tomkins J. L. Kotiaho J. S. Evans J. P. (2012). Comparing evolvabilities: common errors surrounding the calculation and use of coefficients of additive genetic variation. Evolution 66, 2341–2349. 10.1111/j.1558-5646.2011.01565.x 22834736

[B39] George J. P. Grabner M. Campelo F. Karanitsch-Ackerl S. Mayer K. Klumpp R. T.. (2019). Intra-specific variation in growth and wood density traits under water limited conditions: long-term-, short-term-, and sudden responses of four conifer tree species. Sci. Total Environ. 660, 631–643. 10.1016/j.scitotenv.2018.12.478 30641392

[B40] George J. P. Grabner M. Karanitsch-Ackerl S. Mayer K. Weissenbacher L. Schueler S. (2017). Genetic variation, phenotypic stability and repeatability of drought response in European larch throughout 50 years in a common garden experiment. Tree Physiol. 37, 33–46. 10.1093/treephys/tpw085 28173601 PMC5412072

[B41] George J. P. Schueler S. Karanitsch-Ackerl S. Mayer K. Klumpp R. T. Grabner M. (2015). Inter- and intra-specific variation in drought sensitivity in *Abies* spec. and its relation to wood density and growth traits. Agric. For. Meteorol. 214–215, 430–443. 10.1016/j.agrformet.2015.08.268 27713591 PMC5049588

[B42] Gilmour A. R. Gogel B. J. Cullis B. R. Thompson R. (2009). ASReml User Guide Release 3.0. Hemel Hempstead: VSN International Ltd.

[B43] Hanewinkel M. Cullmann D. A. Schelhaas M. J. Nabuurs G.-J. Zimmermann N. E. (2012). Climate change may cause severe loss in the economic value of European forest land. Nat. Clim. Chang. 3, 203–207. 10.1038/nclimate1687

[B44] Hannerz M. Ericsson T. (2007). Planter's guide - a decision support system for the choice of reforestation material, in Seed Orchard. Proceedings from a Conference at Umeå Skogforsk, ed Lindgren E. (Umeå: Skogforsk), 88–94.

[B45] Hannerz M. Sonesson J. Ekberg I. (1999). Genetic correlations between growth and growth rhythm observed in a short term test and performance in long-term field trials of Norway spruce. Canad. J. For. Res. 29, 768–778. 10.1139/x99-056

[B46] Hansen T. F. Pélabon C. Houle D. (2011). Heritability is not evolvability. Evol. Biol. 38:258. 10.1007/s11692-011-9127-6

[B47] Haslinger K. Schöner W. Anders I. (2016). Future drought probabilities in the Greater Alpine Region based on COSMO-CLM experiments – spatial patterns and driving forces. Meteorol. Z. 25, 137–148. 10.1127/metz/2015/0604

[B48] Hijmans R. J. Cameron S. E. Parra J. L. Jones P. G. Jarvis A. (2005). Very high resolution interpolated climate surfaces for global land areas. Int. J. Climatol. 25, 1965–1978. 10.1002/joc.1276

[B49] Hochberg U. Rockwell F. E. Holbrook N. M. Cochard H. (2018). Iso/Anisohydry: a plant-environment interaction rather than a simple hydraulic trait. Trends Plant Sci. 23, 112–120. 10.1016/j.tplants.2017.11.002 29223922

[B50] Houle D. (1992). Comparing evolvability and variability of quantitative traits. Genetics 130, 195–204. 10.1093/genetics/130.1.195 1732160 PMC1204793

[B51] Howe G. T. Aitken S. N. Neale D. B. Jermstad K. D. Wheeler N. C. Chen T. H. H. (2003). From genotype to phenotype: unraveling the complexities of cold adaptation in forest trees. Canad. J. Bot. 81, 1247–1266. 10.1139/b03-141

[B52] Isaac-Renton M. Montwé D. Hamann A. Spiecker H. Cherubini P. Treydte K. (2018). Northern forest tree populations are physiologically maladapted to drought. Nat. Commun. 9:5254. 10.1038/s41467-018-07701-0 30531998 PMC6288165

[B53] Jamnická G. Fleischer P. Jr. Konôpková A. Pšidová E. Kučerová J. Kurjak D.. (2019). Norway spruce (*Picea abies* L.) provenances use different physiological strategies to cope with water deficit. Forests 10:651. 10.3390/f10080651

[B54] Jandl R. Bauhus J. Bolte A. Schindlbacher A. Schueler S. (2015). Effect of climate-adapted forest management on carbon pools and greenhouse gas emissions. Curr. Forest. Rep. 1, 1–7. 10.1007/s40725-015-0006-8

[B55] Johnsen K. H. Flanagan L. B. Huber D. A. Major J. E. (1999). Genetic variation in growth, carbon isotope discrimination, and foliar N concentration in *Picea mariana*: analyses from a half-diallel mating design using field-grown trees. Canad. J. For. Res. 29, 1727–1735. 10.1139/x99-144

[B56] Kapeller S. Dieckmann U. Schueler S. (2017). Varying selection differential throughout the climatic range of Norway spruce in Central Europe. Evol. Appl. 10, 25-38. 10.1111/eva.12413 28035233 PMC5192884

[B57] Kleinschmit J. Bastien J. C. (1992). IUFRO's role in Douglas-fir (*Pseudotsuga menziesii* (Mirb.)Franco) tree improvement. Sil. Genet. 41, 161–173.

[B58] Klisz M. Buras A. Sass-Klaassen U. Puchałka R. Koprowski M. Ukalska J. (2019). Limitations at the limit? Diminishing of genetic effects in Norway spruce provenance trials. Front. Plant Sci. 10:306. 10.3389/fpls.2019.00306 30930924 PMC6425888

[B59] Kurz W. A. Stinson G. Rampley G. J. Dymond C. C. Neilson E. T. (2008). Risk of natural disturbances makes future contribution of Canada's forests to the global carbon cycle highly uncertain. Proc. Natl. Acad. Sci. U.S.A. 105, 1551–1555. 10.1073/pnas.0708133105 18230736 PMC2234182

[B60] Lévesque M. Rigling A. Bugmann H. Weber P. Brang P. (2014). Growth response of five co-occurring conifers to drought across a wide climatic gradient in Central Europe. Agric. For. Meteorol. 197, 1–12. 10.1016/j.agrformet.2014.06.001

[B61] Lévesque M. Saurer M. Siegwolf R. Eilmann B. Brang P. Bugmann H.. (2013). Drought response of five conifer species under contrasting water availability suggests high vulnerability of Norway spruce and European larch. Glob. Chang. Biol. 19, 3184–3199. 10.1111/gcb.12268 23712589

[B62] Lloret F. Keeling E. G. Sala A. (2011). Components of tree resilience: effects of successive low-growth episodes in old ponderosa pine forests. Oikos 120, 1909–1920. 10.1111/j.1600-0706.2011.19372.x

[B63] Marguerit E. Bouffier L. Chancerel E. Costa P. Lagane F. Guehl J.-M.. (2014). The genetics of water-use efficiency and its relation to growth in maritime pine. J. Exp. Bot. 65, 4757–4768. 10.1093/jxb/eru226 24987014 PMC4144764

[B64] Martinez-Meier A. Sanchez L. Pastorino M. Gallo L. Rozenberg P. (2008b). What is hot in tree rings? The wood density of surviving Douglas-firs to the 2003 drought and heat wave. For. Ecol. Manage. 256, 837–843. 10.1016/j.foreco.2008.05.041

[B65] Martinez-Meier A. G. Sanchez L. DallaSalda G. Pastorino M. J. M. Gautry J.-Y. Gallo L. A.. (2008a). Genetic control of the tree-ring response of Douglas-fir (*Pseudotsuga menziesii* (Mirb.)Franco) to the 2003 drought and heat-wave in France. Ann. For. Sci. 65:102. 10.1051/forest:2007074

[B66] Martínez-Sancho E. Rellstab C. Guillaume F. Bigler C. Fonti P. Wohlgemuth T.. (2021). Post-glacial re-colonization and natural selection have shaped growth responses of silver fir across Europe. Sci. Total Environ. 779:146393. 10.1016/j.scitotenv.2021.146393 34030256

[B67] Martin-St. Paul N. Delzon S. Cochard C. (2017). Plant resistance to drought depends on timely stomatal closure. Ecol. Lett. 20, 1437–1447. 10.1111/ele.12851 28922708

[B68] McKee T. B. N. Doesken J. Kleist J. (1993). The relationship of drought frequency and duration to time scales, in Proceedings of Ninth Conference on Applied Climatology (Boston, MA: American Meteorological Society), 179–184.

[B69] Merilä J. Sheldon B. C. (2000). Lifetime reproductive success and heritability in nature. Am. Natur. 155, 301–310. 10.1086/303330 10718727

[B70] Mihai G. Teodosiu M. Birsan M.-V. Alexandru A.-M. Mirancea I. Apostol E.-N.. (2020). Impact of climate change and adaptive genetic potential of Norway spruce at the South–Eastern range of species distribution. Agric. For. Meteorol. 291:108040. 10.1016/j.agrformet.2020.108040

[B71] Montwe D. Spiecker H. Hamann A. (2015). Five decades of growth in a genetic field trial of Douglas-fir reveal trade-offs between productivity and drought tolerance. Tree Genet. Genomes 11:29. 10.1007/s11295-015-0854-1

[B72] Moser E. B. Saxton A. M. Pezeshki S. R. (1990). Repeated measure analysis of variance: application to tree research. Canad. J. For. Res. 20, 524–535. 10.1139/x90-069

[B73] Nakagawa S. Schielzeth H. (2010). Repeatability for Gaussian and non-Gaussian data: a practical guide for biologists. Biol. Rev. 85, 935–956. 10.1111/j.1469-185X.2010.00141.x 20569253

[B74] NDMC (2014). SPI Generator. A Program to Calculate Standardized Precipitation Index. National Drought Mitigation Centre.

[B75] Nobilis F. Godina R. (2006). Extreme Trockenheit in Österreich. Österreich. Wasser Abfallwirt. 58, 51–58. 10.1007/BF03165684

[B76] Payne J. L. Wagner A. (2019). The causes of evolvability and their evolution. Nat. Rev. Genet. 20, 24–38. 10.1038/s41576-018-0069-z 30385867

[B77] Pretzsch H. Schütze G. Uhl E. (2013). Resistance of European tree species to drought stress in mixed versus pure forests: evidence of stress release by inter-specific facilitation. Plant Biol. 15, 483–495. 10.1111/j.1438-8677.2012.00670.x 23062025

[B78] R Development Core Team (2008). R: A Language and Environment for Statistical Computing. Vienna: R Foundation for Statistical Computing.

[B79] Rose L. Leuschner C. Köckemann B. Buschmann H. (2009). Are marginal beech (*Fagus sylvatica* L.) provenances a source for drought tolerant ecotypes? Eur. J. For. Res. 128, 335–343. 10.1007/s10342-009-0268-4

[B80] Rosner S. Klein A. Müller U. Karlsson B. (2007). Hydraulic and mechanical properties of young Norway spruce clones related to growth and wood structure. Tree Physiol. 27, 1165–1178. 10.1093/treephys/27.8.1165 17472942 PMC3197722

[B81] Rosner S. Klein A. Müller U. Karlsson B. (2008). Tradeoffs between hydraulic and mechanical stress response of mature Norway spruce trunkwood. Tree Physiol. 28, 1179–1188. 10.1093/treephys/28.8.1179 18519249 PMC3196968

[B82] Rosner S. Světlík J. Andreassen K. Børja I. Dalsgaard L. Dalsgaard L.. (2014). Wood density as a screening trait for drought sensitivity in Norway spruce. Canad. J. For. Res. 44, 154–161. 10.1139/cjfr-2013-0209

[B83] Rozenberg P. Van Loo J. Hannrup B. Grabner M. (2002). Clonal variation for wood density record of cambium reaction to water deficit in *Picea abies* (L.). Karst. Ann. For. Sci. 59, 533–540. 10.1051/forest:2002038

[B84] Sala A. Piper F. Hoch G. (2010). Physiological mechanisms of drought-induced tree mortality are far from being resolved. New Phytol. 186, 274–281. 10.1111/j.1469-8137.2009.03167.x 20409184

[B85] Sarris D. Christodoulakis D. Körner C. (2007). Recent decline in precipitation and tree growth in the eastern Mediterranean. Glob. Chang. Biol. 13, 1187–1200. 10.1111/j.1365-2486.2007.01348.x

[B86] Schroeder T. A. Hamann A. Wang T. Coops N. C. (2010). Occurrence and dominance of six Pacific Northwest conifer species. J. Veget. Sci. 21, 586–596. 10.1111/j.1654-1103.2009.01163.x

[B87] Schueler S. Falk W. Koskela J. Lefèvre F. Bozzano M. Hubert J.. (2014). Vulnerability of dynamic genetic conservation units of forest trees in Europe to climate change. Glob. Chang. Biol. 20, 1498–1511. 10.1111/gcb.12476 24273066

[B88] Sohn J. A. Ammer C. Bauhus J. Häberle K.-H. Matyssek R. Grams T. E. E. (2013). Mitigation of drought by thinning: short-term and long-term effects on growth and physiological performance of Norway spruce (*Picea abies*). For. Ecol. Manage. 308, 188–197. 10.1016/j.foreco.2013.07.048

[B89] Sohn J. A. Saha S. Bauhus J. (2016). Potential of forest thinning to mitigate drought stress: a meta-analysis. For. Ecol. Manage. 380, 261–273. 10.1016/j.foreco.2016.07.046

[B90] Sokal R. R. Rohlf F. J. (1995). Biometry: The Principles and Practice of Statistics in Biological Research. New York, NY: W. H. Freeman and Company.

[B91] St. Clair J. B. (2006). Genetic variation in fall cold hardiness in coastal Douglas-fir in western Oregon and Washington. Canad. J. Bot. 84, 1110–1121. 10.1139/b06-084

[B92] Stojnic S. Suchocka M. Benito-Garzón M. Torres-Ruiz J. M. Cochard H. Bolte A.. (2018). Variation in xylem vulnerability to embolism in European beech from geographically marginal populations. Tree Physiol. 38, 173–185. 10.1093/treephys/tpx128 29182720

[B93] Suvanto S. Nöjd P. Henttonen H. M. Beuker E. Mäkinen H. (2016). Geographical patterns in the radial growth response of Norway spruce provenances to climatic variation. Agric. For. Meteorol. 222, 10–20. 10.1016/j.agrformet.2016.03.003

[B94] Thoen M. P. Davila Olivas N. H. Kloth K. J. Coolen S. Huang P. P. Aarts M. G. M.. (2017). Genetic architecture of plant stress resistance: multi-trait genome-wide association mapping. New Phytol. 213, 1346–1362. 10.1111/nph.14220 27699793 PMC5248600

[B95] Tollefsrud M. M. Kissling R. Gugerli F. Johnsen Ø. Skrøppa T. Cheddadi R.. (2008). Genetic consequences of glacial survival and postglacial colonization in Norway spruce: combined analysis of mitochondrial DNA and fossil pollen. Mol. Ecol. 17, 4134–4150. 10.1111/j.1365-294X.2008.03893.x 19238710

[B96] Trujillo-Moya C. George J. P. Fluch S. Geburek T. Grabner M. Karanitsch-Ackerl S.. (2018). Drought sensitivity of Norway spruce at the species' warmest fringe: quantitative and molecular analysis reveals high genetic variation among and within provenances. G3 (Bethesda) 8, 1225–1245. 10.1534/g3.117.300524 29440346 PMC5873913

[B97] Vitali V. Büntgen U. Bauhus J. (2017). Silver fir and Douglas fir are more tolerant to extreme droughts than Norway spruce in south-western Germany. Glob. Change Biol. 23, 5108–5119. 10.1111/gcb.13774 28556403

[B98] Wermelinger B. Rigling A. Schneider Mathis D. Dobbertin M. (2008). Assessing the role of bark- and wood-boring insects in the decline of Scots pine (*Pinus sylvestris*) in the Swiss Rhone valley. Ecol. Entomol. 33, 239–249. 10.1111/j.1365-2311.2007.00960.x

[B99] Zang C. Hartl-Meier C. Dittmar C. Rothe A. Menzel A. (2014). Patterns of drought tolerance in major European temperate forest trees: climatic drivers and levels of variability. Glob. Change Biol. 20, 3767–3779. 10.1111/gcb.12637 24838398

