# Supplementary material for: Evolvability of Drought Response in Four Native and Non-native Conifers: Opportunities for Forest and Genetic Resource Management in Europe
Source: Front Plant Sci. 2021 Jul 8;12:648312. doi: 10.3389/fpls.2021.648312 (PMC8295755; doi:10.3389/fpls.2021.648312)
Supplement: Supplementary file 1 [file Data_Sheet_1.docx]

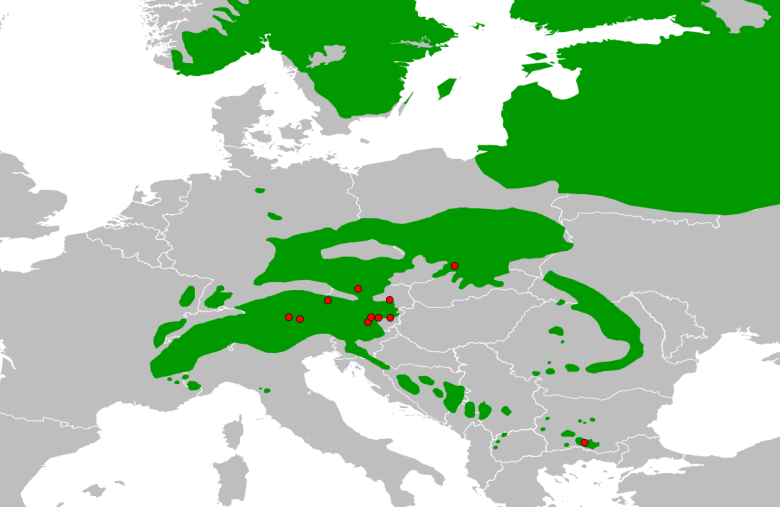

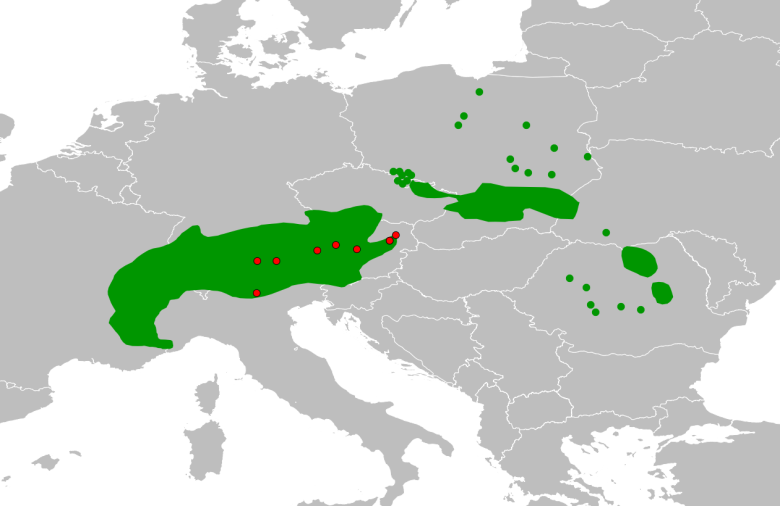

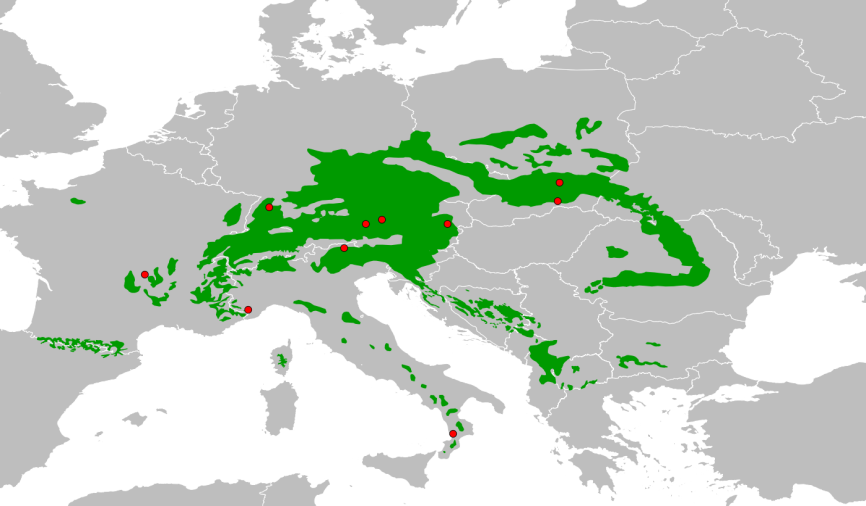
L
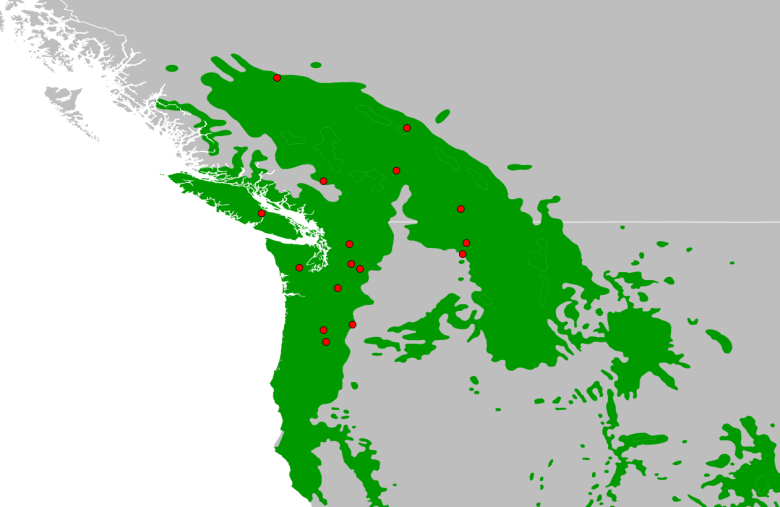


*Pseudotsuga menziesii*

*Picea abies*

*Abies alba*

*Larix decidua*

**Suppl. Fig. S1:** Provenance origin of the tested and sampled provenances and species distributions according to EUFORGEN.

**Suppl. Table S1:** Origin and climate of the tested provenances

| Species | Label | Provenance | Country / State | N | Altitude | Latitude | Longitude | MAT (°C) | APS (mm / year) | maxTWM (°C) | PDM (mm) |
| --- | --- | --- | --- | --- | --- | --- | --- | --- | --- | --- | --- |
| L. decidua | L 01 | Hintersee | AT | 15 | 950 | 47,700 | 13,283 | 6,1 | 1393 | 21,3 | 80 |
| L. decidua | L 02 | Meiselberg-Lammerau | AT | 15 | 700 | 48,059 | 15,920 | 7,1 | 785 | 22,7 | 39 |
| L. decidua | L 03 | Hamet-Lammerau | AT | 15 | 600 | 48,107 | 15,931 | 7,4 | 765 | 23 | 38 |
| L. decidua | L 04 | Hollenstein Seeau | AT | 15 | 950 | 47,750 | 14,767 | 5,4 | 1082 | 21 | 57 |
| L. decidua | L 06 | Kreith Tirol | AT | 15 | 1100 | 47,300 | 11,717 | 6,4 | 937 | 21,6 | 43 |
| L. decidua | L 09 | Miemingerboden | AT | 15 | 850 | 47,300 | 10,983 | 7,3 | 937 | 21,7 | 47 |
| L. decidua | L 10 | Viechtwang Ziehberg | AT | 14 | 500 | 47,917 | 13,967 | 7,7 | 1182 | 23,5 | 72 |
| L. decidua | L 11 | Le Sarche Südtirol | IT | 15 | 900 | 46,067 | 10,967 | 8,6 | 786 | 24,3 | 34 |
| A. alba | 14 | Brixen-Vintl | IT | 16 | 1100 | 46°49' | 11°43' | 7,9 | 827 | 24,1 | 34 |
| A. alba | 19 | Stara-Voda | SK | 9 | 700 | 48°48' | 20°41' | 6,9 | 676 | 22,8 | 31 |
| A. alba | 22 | Nawojowa | PL | 10 | 600 | 49°34' | 20°45' | 7,9 | 735 | 24,2 | 31 |
| A. alba | 34 | Siegsdorf | DE | 15 | 900 | 47°49' | 12°38' | 7,5 | 1108 | 22,8 | 57 |
| A. alba | 39 | Serra San Bruno | IT | 10 | 1100-1400 | 38°59' | 16°18' | 15,8 | 947 | 28,2 | 17 |
| A. alba | 45 | Valle Pesio | IT | 12 | 1200-1400 | 44°14' | 07°40' | 8,9 | 897 | 21,6 | 43 |
| A. alba | 88 | Hohe Wand | AT | 12 | 700-900 | 47°50' | 16°03' | 7,6 | 767 | 23,3 | 37 |
| A. alba | 90 | Schneegattern | AT | 10 | 550-900 | 48°01' | 13°18' | 7,9 | 1257 | 23,3 | 75 |
| A. alba | 113 | Pfalzgrafenweiler | DE | 17 | 700 | 48°32' | 08°34' | 8,2 | 949 | 22,5 | 64 |
| A. alba | 122 | Maubert | FR | 15 | 750-850 | 45°42' | 03°21' | 10,4 | 649 | 25 | 32 |
| P. abies | b03 | Ridelov | CZ | 27 | 650 | 49,23 | 15,4 | 6,7 | 717 | 21,6 | 37 |
| P. abies | d04 | Istebna | PL | 34 | 600 | 49,578 | 18,913 | 6,7 | 955 | 22,2 | 47 |
| P. abies | d14 | Tschepelare | BG | 30 | 1350 - 1500 | 41,736 | 24,669 | 6,2 | 710 | 21,3 | 38 |
| P. abies | I19 | Innsbruck | AT | 22 | 400 - 900 | 47,315 | 11,563 | 7,6 | 926 | 22,9 | 43 |
| P. abies | Q14 | Lankowitz | AT | 32 | 600 | 47,082 | 15,075 | 7,6 | 940 | 24,2 | 38 |
| P. abies | R06 | Hintergams | AT | 23 | 900 - 1300 | 47,317 | 15,203 | 3,3 | 1173 | 18,1 | 60 |
| P. abies | R13 | Hohenau | AT | 22 | 400 - 900 | 47,275 | 15,53 | 6,5 | 927 | 22,4 | 38 |
| P. abies | S10 | Klausen-Leopoldsdorf | AT | 24 | 500 | 48,078 | 16,024 | 8,4 | 707 | 24,2 | 35 |
| P. abies | ST | Schneegattern | AT | 55 | 400 - 600 | 48,042 | 13,287 | 7,6 | 1259 | 23 | 75 |
| P. abies | X05 | Rosenhof-Sandl | AT | 27 | 800 - 850 | 48,582 | 14,64 | 5,5 | 997 | 20,5 | 58 |
| P. abies | Y18 | Hartberg | AT | 27 | 300 - 600 | 47,283 | 16,041 | 8,5 | 780 | 24,5 | 32 |
| P. menziesii | D10 | D'Arcy - 1021 | CA/BC | 13 | 250 | 51 | -123 | 5,7 | 641 | 24 | 31 |
| P. menziesii | D15 | Nelson - 1035 | CA/BC | 13 | 750-900 | 50 | -117 | 7,1 | 776 | 26 | 44 |
| P. menziesii | D16 | Alberni - 1036 | CA/BC | 10 | 150 | 49 | -125 | 8,7 | 1789 | 23 | 40 |
| P. menziesii | D19 | Newport - 1055 | US/WA | 11 | 750 | 48 | -117 | 7,6 | 687 | 29 | 26 |
| P. menziesii | D20 | Spokane - 1065 | US/WA | 11 | 550-650 | 48 | -117 | 8,1 | 568 | 29 | 21 |
| P. menziesii | D23 | Matlock - 1076 | US/WA | 11 | 100 | 47 | -123 | 9,8 | 2278 | 24 | 38 |
| P. menziesii | D24 | Cle Elum - 1078 | US/WA | 9 | 650 | 47 | -121 | 7,3 | 1056 | 25 | 19 |
| P. menziesii | D25 | Pine Grove - 1099 | US/OR | 10 | 750 | 45 | -121 | 8,5 | 747 | 28 | 12 |
| P. menziesii | D26 | Fort St.James - 1106 | CA/BC | 10 | 850 | 54 | -124 | 1,6 | 505 | 21 | 21 |
| P. menziesii | D28 | Clemina - 1110 | CA/BC | 11 | 900 | 53 | -119 | 0,3 | 791 | 18 | 43 |
| P. menziesii | D33 | Randle | US/WA | 13 | 350 - 500 | 46 | -122 | 9,0 | 1835 | 25 | 30 |
| P. menziesii | D37 | Darrington | US/WA | 15 | 950 - 1.100 | 48 | -121 | 4,9 | 2028 | 20 | 46 |
| P. menziesii | D38 | Adams Lake | CA/BC | 11 | 500 - 650 | 51 | -120 | 5,8 | 499 | 26 | 27 |
| P. menziesii | D39 | Abiqua Basin | US/OR | 11 | 600 - 750 | 45 | -123 | 8,8 | 2161 | 23 | 28 |
| P. menziesii | D40 | Cascadia | US/OR | 10 | 650 - 800 | 44 | -122 | 7,9 | 2210 | 24 | 26 |
| P. menziesii | D44 | Snoqualmie - Pass | US/WA | 12 | 650 - 800 | 47 | -121 | 6,9 | 1978 | 22 | 39 |
| MAT -Mean Annual Temperature | | |  |  |  |  |  |  |  |  |  |
| APS - Annual Precipitation Sum | | |  |  |  |  |  |  |  |  |  |
| maxTWM - Max. Temperature of Warmest Month | | | |  |  |  |  |  |  |  |  |
| PDM - Precipitation of Driest Months | | |  |  |  |  |  |  |  |  |  |

**Suppl. Table S2:** Coincidence of drought events at the four trial sites as given by Pearson’s correlation coefficients R between the SPI-times series. Above diagonal: correlations of SPI for drought periods of one month; below diagonal: correlations of SPI for drought periods of three months. AA - *Abies alba*; PA - *Picea abies*; LD - *Larix decidua*; PM - *Pseudotsuga menziesii*.

|  | SPI-LD | SPI-PM | SPI-PA | SPI-AA |
| --- | --- | --- | --- | --- |
| SPI-LD | - | 0.751 | 0.940 | 0.845 |
| SPI-PM | 0.772 | - | 0.811 | 0.787 |
| SPI-PA | 0.933 | 0.812 | - | 0.860 |
| SPI-AA | 0.819 | 0.745 | 0.807 | - |

**Suppl. Table S3:** Repeatability and estimated standard error of drought response for individual provenances of Norway spruce. Numbers in bold indicate significant repeatability values; V_es_, general environmental variance; V_res_, residual variance; r, repeatability, V_pro_, variance arising due to the incorporation of ‘provenance’ as a covariate; N.E., no estimate.

| Provenance | Measure | Resistance | Recovery |
| --- | --- | --- | --- |
| b03 | V_es_ | 0.011 ± 0.088 | 0.043 ± 0.114 |
|  | V_res_ | 0.737 ± 0.142 | 0.926 ± 0.178 |
|  | r | 0.015 ± 0.114 | 0.045 ± 0.117 |
|  |  |  |  |
| d4 | V_es_ | 0.000 ± 0.000 | 0.000 ± 0.000 |
|  | V_res_ | 0.929 ± 0.131 | 0.819 ± 0.115 |
|  | r | 0.000 ± 0.000 | N.E. |
|  |  |  |  |
| R6 | V_es_ | 0.198 ± 0.133 | 0.035 ± 0.077 |
|  | V_res_ | 0.650 ± 0.135 | 0.557 ± 0.116 |
|  | r | **0.233 ± 0.137** | 0.059 ± 0.129 |
|  |  |  |  |
| ST | V_es_ | 0.106 ± 0.110 | 0.404 ± 0.143 |
|  | V_res_ | 1.172 ± 0.158 | 0.922 ± 0.124 |
|  | r | 0.083 ± 0.084 | **0.305 ± 0.088** |
|  |  |  |  |
| X5 | V_es_ | 0.071 ± 0.120 | 0.013 ± 0.078 |
|  | V_res_ | 0.928 ± 0.178 | 0.664 ± 0.128 |
|  | r | 0.071 ± 0.119 | 0.019 ± 0.115 |
|  |  |  |  |
| d14 | V_es_ | 0.304 ± 0.135 | 0.138 ± 0.092 |
|  | V_res_ | 0.575 ± 0.105 | 0.560 ± 0.102 |
|  | r | **0.346 ± 0.118** | **0.198 ± 0.119** |
|  |  |  |  |
| I19 | V_es_ | 0.151 ± 0.121 | 0.219 ± 0.142 |
|  | V_res_ | 0.636 ± 0.136 | 0.648 ± 0.138 |
|  | r | **0.192 ± 0.140** | **0.253 ± 0.141** |
|  |  |  |  |
| Q14 | V_es_ | 0.373 ± 0.148 | 0.149 ± 0.116 |
|  | V_res_ | 0.584 ± 0.103 | 0.802 ± 0.142 |
|  | r | **0.389 ± 0.112** | **0.157 ± 0.114** |
|  |  |  |  |
| R13 | V_es_ | 0.517 ± 0.232 | 0.162 ± 0.216 |
|  | V_res_ | 0.657 ± 0.140 | 1.388 ± 0.296 |
|  | r | **0.440 ± 0.132** | 0.105 ± 0.135 |
|  |  |  |  |
| S10 | V_es_ | 0.381 ± 0.171 | 0.239 ± 0.124 |
|  | V_res_ | 0.552 ± 0.113 | 0.498 ± 0.102 |
|  | r | **0.408 ± 0.129** | **0.324 ± 0.133** |
|  |  |  |  |
| Y18 | V_es_ | 0.061 ± 0.112 | 0.000 ± 0.000 |
|  | V_res_ | 0.872 ± 0.168 | 1.155 ± 0.183 |
|  | r | 0.065 ± 0.119 | N.E. |
|  |  |  |  |
| Overall | V_pro_ | 0.022 ± 0.018 | 0.032 ± 0.021 |
|  | V_es_ | 0.182 ± 0.039 | 0.129 ± 0.036 |
|  | V_res_ | 0.798 ± 0.044 | 0.838 ± 0.047 |
|  | r | **0.182 ± 0.036** | **0.129 ± 0.035** |
|  |  |  |  |
|  |  |  |  |

**Suppl. Table S4:** Repeatability and estimated standard error of drought response for individual provenances of Douglas-fir. Numbers in bold indicate significant repeatability values; V_es_, general environmental variance; V_res_, residual variance; r, repeatability, V_pro_, variance arising due to the incorporation of ‘provenance’ as a covariate; N.E., no estimate.

| Provenance | Measure | Resistance | Recovery |
| --- | --- | --- | --- |
| 10 | V_es_ | 0.000 ± 0.000 | 0.000 ± 0.000 |
| 10 | V_res_ | 1.124 ± 0.258 | 0.952 ± 0.218 |
| 10 | r | 0.000 ± 0.000 | 0.000 ± 0.000 |
|  |  |  |  |
| 15 | V_es_ | 0.000 ± 0.000 | 0.000 ± 0.000 |
| 15 | V_res_ | 1.553 ± 0.356 | 1.549 ± 0.355 |
| 15 | r | N.E. | 0.000 ± 0.000 |
|  |  |  |  |
| 16 | V_es_ | 0.071 ± 0.105 | 0.203 ± 0.212 |
| 16 | V_res_ | 0.402 ± 0.127 | 0.657 ± 0.208 |
| 16 | r | 0.149 ± 0.209 | **0.236 ± 0.213** |
|  |  |  |  |
| 19 | V_es_ | 0.000 ± 0.000 | 0.105 ± 0.214 |
| 19 | V_res_ | 1.113 ± 0.278 | 0.959 ± 0.289 |
| 19 | r | 0.000 ± 0.000 | 0.099 ± 0.194 |
|  |  |  |  |
| 20 | V_es_ | 0.000 ± 0.000 | 0.069 ± 0.137 |
| 20 | V_res_ | 0.853 ± 0.213 | 0.622 ± 0.187 |
| 20 | r | N.E. | 0.100 ± 0.194 |
|  |  |  |  |
| 23 | V_es_ | 0.000 ± 0.000 | 0.166 ± 0.182 |
| 23 | V_res_ | 0.648 ± 0.162 | 0.640 ± 0.193 |
| 23 | r | N.E. | **0.206 ± 0.202** |
|  |  |  |  |
| 24 | V_es_ | 0.000 ± 0.000 | 0.000 ± 0.000 |
| 24 | V_res_ | 1.400 ± 0.388 | 1.328 ± 0.368 |
| 24 | r | 0.000 ± 0.000 | 0.000 ± 0.000 |
|  |  |  |  |
| 25 | V_es_ | 0.001 ± 0.099 | 0.118 ± 0.148 |
| 25 | V_res_ | 0.498 ± 0.158 | 0.516 ± 0.163 |
| 25 | r | 0.002 ± 0.190 | 0.187 ± 0.211 |
|  |  |  |  |
| 26 | V_es_ | 0.000 ± 0.000 | 0.176 ± 0.255 |
| 26 | V_res_ | 1.665 ± 0.437 | 0.969 ± 0.307 |
| 26 | r | 0.000 ± 0.000 | 0.154 ± 0.209 |
|  |  |  |  |
| 28 | V_es_ | 0.000 ± 0.000 | 0.000 ± 0.000 |
| 28 | V_res_ | 1.623 ± 0.406 | 1.977 ± 0.494 |
| 28 | r | 0.000 | N.E. |
|  |  |  |  |
| 33 | V_es_ | 0.000 ± 0.000 | 0.000 ± 0.000 |
| 33 | V_res_ | 0.718 ± 0.165 | 1.032 ± 0.237 |
| 33 | r | 0.000 ± 0.000 | 0.000 ± 0.000 |
|  |  |  |  |
| 37 | V_es_ | 0.000 ± 0.000 | 0.000 ± 0.000 |
| 37 | V_res_ | 0.543 ± 0.116 | 0.858 ± 0.183 |
| 37 | r | N.E. | 0.000 ± 0.000 |
|  |  |  |  |
| 38 | V_es_ | 0.000 ± 0.000 | 0.081 ± 0.224 |
| 38 | V_res_ | 1.345 ± 0.336 | 1.080 ± 0.325 |
| 38 | r | 0.000 ± 0.000 | 0.070 ± 0.190 |
|  |  |  |  |
| 39 | V_es_ | 0.125 ± 0.154 | 0.101 ± 0.118 |
| 39 | V_res_ | 0.569 ± 0.175 | 0.423 ± 0.130 |
| 39 | r | 0.180 ± 0.204 | 0.193 ± 0.204 |
|  |  |  |  |
| 40 | V_es_ | 0.000 ± 0.000 | 0.041 ± 0.120 |
| 40 | V_res_ | 0.955 ± 0.251 | 0.551 ± 0.174 |
| 40 | r | N.E. | 0.069 ± 0.200 |
|  |  |  |  |
| 44 | V_es_ | 0.000 ± 0.000 | 0.000 ± 0.000 |
| 44 | V_res_ | 0.666 ± 0.159 | 0.668 ± 0.160 |
| 44 | r | N.E. | N.E. |
|  |  |  |  |
| Overall | V_pro_ | 0.148 ± 0.164 | 0.007 ± 0.014 |
|  | V_es_ | 0.000 ± 0.000 | 0.000 ± 0.000 |
|  | V_res_ | 0.983 ± 0.061 | 0.990 ± 0.061 |
|  | r | 0.000 ± 0.000 | 0.000 ± 0.000 |

**Suppl. Table S5:** Repeatability and estimated standard error of drought response for individual provenances of silver fir. Numbers in bold indicate significant repeatability values; V_es_, general environmental variance; V_res_, residual variance; r, repeatability, V_pro_, variance arising due to the incorporation of ‘provenance’ as a covariate; N.E., no estimate.

| Provenance | Measure | Resistance | Recovery |
| --- | --- | --- | --- |
| 14 | V_es_ | 0.000 ± 0.000 | 0.060 ± 0.162 |
| 14 | V_res_ | 0.797 ± 0.166 | 0.920 ± 0.235 |
| 14 | r | N.E. | 0.061 ± 0.162 |
| 14 |  |  |  |
| 19 | V_es_ | 0.000 ± 0.000 | 0.000 ± 0.000 |
| 19 | V_res_ | 1.439 ± 0.406 | 1.794 ± 0.507 |
| 19 | r | N.E. | N.E. |
| 19 |  |  |  |
| 22 | V_es_ | 0.055 ± 0.135 | 0.005 ± 0.104 |
| 22 | V_res_ | 0.562 ± 0.187 | 0.480 ± 0.160 |
| 22 | r | 0.090 ± 0.215 | 0.011 ± 0.202 |
| 22 |  |  |  |
| 34 | V_es_ | 0.000 ± 0.000 | 0.000 ± 0.000 |
| 34 | V_res_ | 0.948 ± 0.202 | 0.776 ± 0.166 |
| 34 | r | N.E. | 0.000 ± 0.000 |
| 34 |  |  |  |
| 39 | V_es_ | 0.110 ± 0.194 | 0.000 ± 0.000 |
| 39 | V_res_ | 0.782 ± 0.247 | 0.800 ± 0.210 |
| 39 | r | 0.124 ± 0.207 | 0.000 ± 0.000 |
| 39 |  |  |  |
| 45 | V_es_ | 0.000 ± 0.000 | 0.170 ± 0.210 |
| 45 | V_res_ | 1.039 ± 0.252 | 0.821 ± 0.241 |
| 45 | r | 0.000 ± 0.000 | 0.172 ± 0.195 |
| 45 |  |  |  |
| 88 | V_es_ | 0.000 ± 0.000 | 0.000 ± 0.000 |
| 88 | V_res_ | 0.739 ± 0.179 | 0.442 ± 0.107 |
| 88 | r | N.E. | N.E. |
| 88 |  |  |  |
| 90 | V_es_ | 0.397 ± 0.263 | 0.378 ± 0.235 |
| 90 | V_res_ | 0.451 ± 0.143 | 0.339 ± 0.107 |
| 90 | r | **0.468 ± 0.195** | **0.527 ± 0.184** |
| 90 |  |  |  |
| 113 | V_es_ | 0.123 ± 0.189 | 0.374 ± 0.219 |
| 113 | V_res_ | 1.058 ± 0.257 | 0.678 ± 0.165 |
| 113 | r | 0.104 ± 0.155 | **0.356 ± 0.158** |
| 113 |  |  |  |
| 122 | V_es_ | 0.252 ± 0.204 | 0.000 ± 0.000 |
| 122 | V_res_ | 0.772 ± 0.200 | 1.268 ± 0.270 |
| 122 | r | **0.246 ± 0.172** | N.E. |
|  |  |  |  |
| Overall | V_pro_ | 0.023 ± 0.025 | 0.044 ± 0.034 |
|  | V_es_ | 0.037 ± 0.055 | 0.012 ± 0.053 |
|  | V_res_ | 0.937 ± 0.084 | 0.944 ± 0.085 |
|  | r | 0.037 ± 0.055 | 0.012 ± 0.052 |

**Suppl. Table S6:** Repeatability and estimated standard error of drought response for individual provenances of European larch. Numbers in bold indicate significant repeatability values; V_es_, general environmental variance; V_res_, residual variance; r, repeatability, V_pro_, variance arising due to the incorporation of ‘provenance’ as a covariate; N.E., no estimate.

| Provenance | Measure | Resistance | Recovery |
| --- | --- | --- | --- |
|  | V_es_ | 0.093 ± 0.331 | 0.000 ± 0.000 |
| 1 | V_res_ | 1.720 ± 0.466 | 1.401 ± 0.302 |
|  | r | 0.051 ± 0.179 | N.E. |
|  |  |  |  |
|  | V_es_ | 0.143 ± 0.264 | 0.515 ± 0.296 |
| 2 | V_res_ | 1.415 ± 0.366 | 0.745 ± 0.193 |
|  | r | 0.092 ± 0.164 | **0.409 ± 0.164** |
|  |  |  |  |
|  | V_es_ | 0.070 ± 0.152 | 0.000 ± 0.000 |
| 3 | V_res_ | 0.845 ± 0.218 | 1.050 ± 0.224 |
|  | r | 0.077 ± 0.162 | N.E. |
|  |  |  |  |
|  | V_es_ | 0.218 ± 0.209 | 0.198 ± 0.196 |
| 4 | V_res_ | 0.897 ± 0.232 | 0.848 ± 0.219 |
|  | r | **0.195 ± 0.171** | **0.190 ± 0.171** |
|  |  |  |  |
|  | V_es_ | 0.235 ± 0.161 | 0.291 ± 0.201 |
| 6 | V_res_ | 0.502 ± 0.132 | 0.580 ± 0.155 |
|  | r | **0.319 ± 0.174** | **0.334 ± 0.181** |
|  |  |  |  |
|  | V_es_ | 0.000 ± 0.000 | 0.153 ± 0.144 |
| 9 | V_res_ | 0.706 ± 0.151 | 0.606 ± 0.157 |
|  | r | N.E. | **0.201 ± 0.171** |
|  |  |  |  |
|  | V_es_ | 0.132 ± 0.097 | 0.278 ± 0.162 |
| 10 | V_res_ | 0.311 ± 0.083 | 0.376 ± 0.101 |
|  | r | **0.299 ± 0.177** | **0.425 ± 0.168** |
|  |  |  |  |
|  | V_es_ | 0.085 ± 0.130 | 0.114 ± 0.158 |
| 11 | V_res_ | 0.667 ± 0.172 | 0.792 ± 0.205 |
|  | r | 0.113 ± 0.166 | 0.126 ± 0.167 |
|  |  | 0.093 ± 0.331 |  |
| Overall | V_pro_ | 0.000 ± 0.000 | 0.005 ± 0.019 |
|  | V_es_ | 0.112 ± 0.061 | 0.160 ± 0.065 |
|  | V_res_ | 0.884 ± 0.082 | 0.832 ± 0.077 |
|  | r | **0.112 ± 0.059** | **0.161 ± 0.061** |
